# Genome-matched treatments and patient outcomes in the Maine Cancer Genomics Initiative (MCGI)

**DOI:** 10.1038/s41698-024-00547-4

**Published:** 2024-03-09

**Authors:** Eric C. Anderson, John DiPalazzo, F. Lee Lucas, Michael J. Hall, Andrey Antov, Petra Helbig, Jennifer Bourne, Leah Graham, Lory Gaitor, Christine Lu-Emerson, Leslie S. Bradford, Roger Inhorn, Sarah J. Sinclair, Philip L. Brooks, Christian A. Thomas, Karen Rasmussen, Paul K. J. Han, Edison T. Liu, Jens Rueter

**Affiliations:** 1 Center for Interdisciplinary Population and Health Research, MaineHealth Institute for Research, Portland, ME USA; 2 Tufts University School of Medicine, Boston, MA USA; 3 Fox Chase Cancer Center, Philadelphia, PA USA; 4 The Jackson Laboratory, Augusta, ME USA; 5 MaineHealth Cancer Care, South Portland, ME USA; 6 Maine Medical Partners Women’s Health, Gynecologic Oncology, Scarborough, ME USA; 7 PenBay Medical Center Oncology, Rockport, ME USA; 8 Northern Light Cancer Institute, Brewer, ME USA; 9 New England Cancer Specialists, Scarborough, ME USA; 10 Spectrum Healthcare, South Portland, ME USA; 11 National Cancer Institute, Bethesda, MD USA; 12 The Jackson Laboratory for Genomic Medicine, Farmington, CT USA

**Keywords:** Molecular medicine, Cancer genomics, Tumour biomarkers, Cancer genomics, Outcomes research

## Abstract

Genomic tumor testing (GTT) is an emerging technology aimed at identifying variants in tumors that can be targeted with genomically matched drugs. Due to limited resources, rural patients receiving care in community oncology settings may be less likely to benefit from GTT. We analyzed GTT results and observational clinical outcomes data from patients enrolled in the Maine Cancer Genomics Initiative (MCGI), which provided access to GTTs; clinician educational resources; and genomic tumor boards in community practices in a predominantly rural state. 1603 adult cancer patients completed enrollment; 1258 had at least one potentially actionable variant identified. 206 (16.4%) patients received a total of 240 genome matched treatments, of those treatments, 64% were FDA-approved in the tumor type, 27% FDA-approved in a different tumor type and 9% were given on a clinical trial. Using Inverse Probability of Treatment Weighting to adjust for baseline characteristics, a Cox proportional hazards model demonstrated that patients who received genome matched treatment were 31% less likely to die within 1 year compared to those who did not receive genome matched treatment (HR: 0.69; 95% CI: 0.52–0.90; *p*-value: 0.006). Overall, GTT through this initiative resulted in levels of genome matched treatment that were similar to other initiatives, however, clinical trials represented a smaller share of treatments than previously reported, and "off-label" treatments represented a greater share. Although this was an observational study, we found evidence for a potential 1-year survival benefit for patients who received genome matched treatments. These findings suggest that when disseminated and implemented with a supportive infrastructure, GTT may benefit cancer patients in rural community oncology settings, with further work remaining on providing genome-matched clinical trials.

## Introduction

Genomic tumor testing (GTT) is an emerging technology aimed at identifying variants in tumors that may identify genomically targeted drugs. Testing for specific variants has become standard of care in many cancers (e.g., EGFR analysis in non-small cell lung cancer), but next generation sequencing has enabled the ability to examine hundreds of genes and biomarkers that are implicated in cancer biology. A number of precision oncology initiatives have attempted to translate this promising technology into improved patient outcomes by scaling up testing and supporting clinical decision making. To date, these initiatives are typically based at large academic medical centers in urban areas (e.g., Mi-ONCOSEQ at the Michigan Center for Translational Pathology^1^; MSK-IMPACT at Memorial Sloan Kettering Cancer Center^2^; UC San Diego Moores Cancer Center)^3,4^ or integrated healthcare systems (e.g., Intermountain Healthcare; Levine Cancer Institute, Atrium Health; National Cancer Care Alliance)^5^. Other precision oncology initiatives include tumor-specific programs led by private sequencing companies (e.g., Know Your Tumor program in pancreatic cancer)^6^ and international programs such as the Cancer Molecular Screening and Therapeutics (MoST) in Australia^7^, the Copenhagen Prospective Personalized Oncology study in Denmark^8^ and the Drug Rediscovery Protocol (DRUP) in the Netherlands^9^.

Although promising, outcomes of precision medicine initiatives remain uncertain and dependent on the patient population and initiative^10^. Studies have generally identified clinically actionable genomic alterations in many patients, ranging from 40% to 94%^2,8,11–17^, but usually only 10% to 25% of patients have received therapy informed by GTT^2,3,8^. A number of studies have demonstrated modest clinical benefit^3,8,14,18–22^, especially when treatment choices were guided by a high level of evidence^23^, although it has been challenging to demonstrate benefit in randomized clinical trials across tumor types^24,25^, likely due to clinical trial design challenges^24–26^.

Rural patients receiving care in community oncology settings may be less likely to benefit from GTT. Patients often do not receive treatment concordant with test results for a variety of reasons, including lack of access to clinical trials^27^. Furthermore, rural clinicians report being less likely to have support including on-site genetic counselors, established protocols for genomic testing, and molecular tumor boards for decision support^28^. These and other factors may hinder the availability and utilization of genomic testing and treatment in community oncology practices that serve rural, low resource patients, and might create or exacerbate cancer disparities^29^.

To our knowledge, the outcomes of GTT in community oncology practices that serve primarily rural patients have not been systematically evaluated. To address this knowledge gap, we analyzed clinical outcomes of patients enrolled in the Maine Cancer Genomics Initiative (MCGI), an initiative that provided access to GTT, clinician educational resources, and genomic tumor boards to community practices in a predominantly rural state^30^. We evaluated the characteristics of patients enrolled in the MCGI, the genomic alterations identified, and the therapeutic impact of this initiative.

## Results

### Study population, GTT utilization and diagnostic yield

**Fig. 1:**
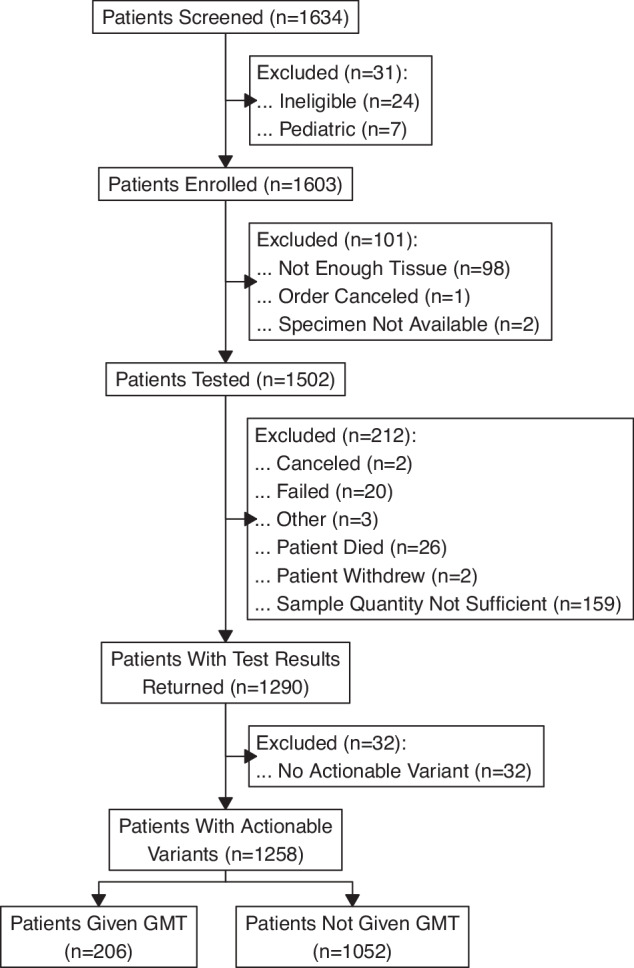
MCGI Consort diagram.

1603 adult patients completed enrollment (Fig. 1). 1502 patients had GTTs attempted by the laboratories, and 1290 had results returned to clinicians (85.9%). The most common reason results were not returned was insufficient quantity or quality of the provided sample. Of the 1290 patients with results returned, the vast majority (*n* = 1258, 97.5%) had at least one potentially actionable variant (based on diagnostic, prognostic or therapeutic criteria) identified. Only 32 patients (2.5%) had no actionable variants identified (meaning either only “variants of unknown significance” or no variants at all were reported). This group of 1258 patients with potentially actionable variants identified were the focus of the analysis below. This sample contained slightly more female patients (60.0%) and had an average age of 63.8 years (range 19–94 years; Table 1). A majority of patients had not completed a college degree (70.8%), came from households with less than $50,000 US dollars annual household income (56.6%), and lived in a rural, non-urban, setting (73.5%). Stage IV (or Grade 4 if brain cancer) was most common (74.0%). Lung (12.9%) and breast (10.3%) malignancies had the highest proportion of all analyzed patients.

**Table 1:** Characteristics of patients with actionable variants

Characteristic	Actionable Variant, GMT Given, *N* = 206	Actionable Variant, No GMT, *N* = 1052	*p*-value^a^
Age, Median (IQR)	64 (57, 71)	65 (57, 72)	0.2
Unknown	24	130	
Sex, *n* (%)			0.8
Female	107 (59%)	555 (60%)	
Male	75 (41%)	367 (40%)	
Unknown	24	130	
Race, *n* (%)			0.2
White	173 (84%)	880 (84%)	
African or African-American	1 (0.5%)	5 (0.5%)	
Asian	1 (0.5%)	3 (0.3%)	
American Indian or Alaskan Native	3 (1.5%)	3 (0.3%)	
Not Given/Other	28 (14%)	147 (14%)	
Multiple	0 (0%)	14 (1.3%)	
Unknown	0	0	
Ethnicity, *n* (%)			>0.9
Hispanic	2 (1.1%)	10 (1.1%)	
Non-Hispanic	176 (99%)	900 (99%)	
Unknown	28	142	
Rurality, *n* (%)			0.6
Metro	49 (28%)	229 (26%)	
Large rural	68 (39%)	311 (36%)	
Small rural	46 (26%)	266 (30%)	
Isolated rural	11 (6.3%)	70 (8.0%)	
Unknown	32	176	
Household income, *n* (%)			0.7
Less than $25,000	50 (30%)	234 (27%)	
$25,000–$49,999	41 (25%)	265 (30%)	
$50,000–$74,999	34 (20%)	152 (17%)	
$75,000–$100,000	15 (9.0%)	81 (9.3%)	
More than $100,000	17 (10%)	89 (10%)	
Don’t know	10 (6.0%)	54 (6.2%)	
Unknown	39	177	
Education, *n* (%)			0.5
Less than high school	9 (5.2%)	60 (6.7%)	
High School Graduate/GED	59 (34%)	280 (31%)	
Some college/Trade School	62 (36%)	285 (32%)	
Bachelor’s or Advanced Degree	44 (25%)	267 (30%)	
Unknown	32	160	
Insurance, *n* (%)			>0.9
Medicare and Medicaid	12 (7.6%)	64 (8.0%)	
Medicare	80 (51%)	424 (53%)	
Medicaid	8 (5.1%)	38 (4.8%)	
Private	58 (37%)	273 (34%)	
Unknown	48	253	
Cancer Stage, *n* (%)^b^			0.004
Stage I	5 (2.5%)	45 (4.4%)	
Stage II	4 (2.0%)	47 (4.6%)	
Stage III	17 (8.6%)	163 (16%)	
Stage IV	172 (87%)	758 (75%)	
Unknown	8	39	
Cancer Site Category, *n* (%)			0.002
Gynecologic	32 (16%)	223 (21%)	
Lung	35 (17%)	124 (12%)	
Breast	29 (14%)	101 (9.6%)	
Colon	14 (6.8%)	123 (12%)	
Brain	19 (9.2%)	113 (11%)	
Prostate	4 (1.9%)	60 (5.7%)	
Other	73 (35%)	307 (29%)	
Unknown	0	1	
Quality of Life, Median (IQR)	45 (40, 51)	45 (40, 51)	0.9
Unknown	30	154	

^a^Wilcoxon rank sum test; Pearson’s Chi-squared test.

^b^Brain cancers rated by Grade, not Stage.

A total of 211 unique variants and biomarkers were identified as potentially actionable (see Supplementary Table 1 for complete list). As expected, TP53 variants were the most frequently observed aberrations (641 patients), followed by PDL1 positivity (318 patients). The next most common variants were in KRAS (233 patients) and APC (166 patients). 60 variants were only identified once, some of them rare but potentially therapeutically actionable (e.g., FGFR3-TACC3 fusion).

### Receipt of genome matched treatment

As described in the methods, a genome matched treatment (GMT) was counted if patients received a drug based on drug-biomarker matches or GTT report matches. Of the 1258 patients for whom at least one actionable variant was identified, 206 (16.4%) received at least one GMT and 1052 did not receive any GMT (Table 1). No differences in age, sex, race/ethnicity, rurality, household income, education or quality of life were identified between the GMT and no GMT groups (Table 1). However, there was a significant difference in cancer stage and site identified between the two groups. Patients with stage IV cancers were more frequently found in the GMT group. With respect to cancer site, patients with lung, breast, gastroesophageal, urinary, and melanoma cancers were more frequently found in the GMT group, while patients with gynecologic, brain, colon, pancreas, prostate and other cancer sites were more common in the non-GMT group. With respect to the method of ascertainment of GMT, of the 206 patients identified as having received at least one GMT, 65 patients were matched only on FDA label (i.e., these patients had no matches via the test report). 59 patients were matched only on recommendations from the GTT report, and 82 patients were matched on both FDA labels and GTT reports (Supplementary Table 2).

**Fig. 2:**
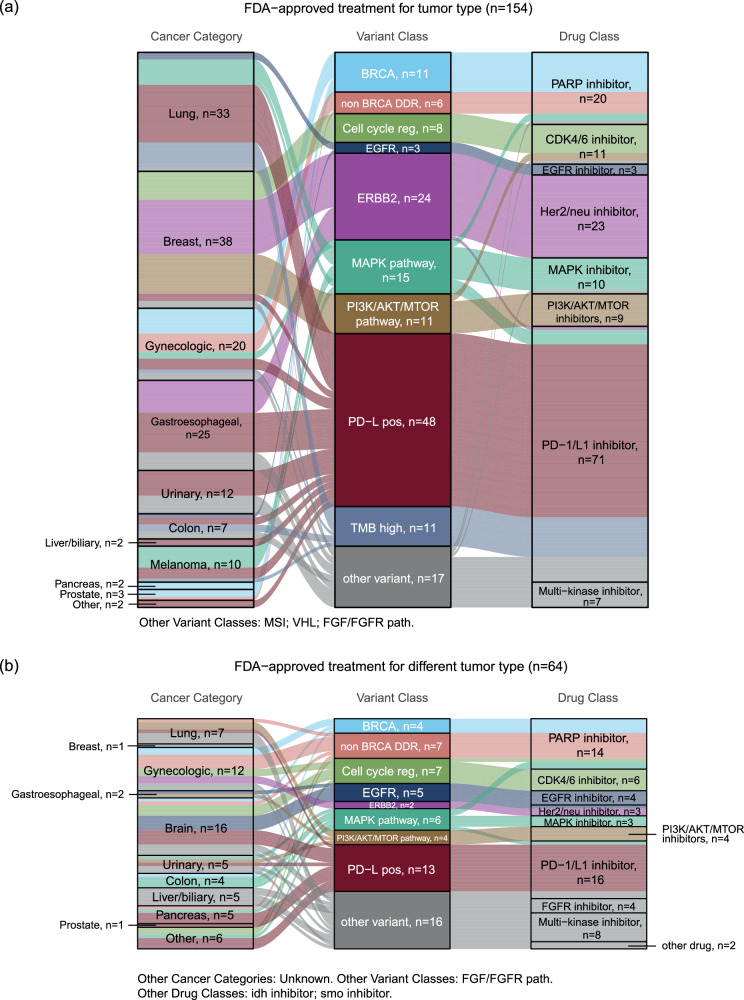
Cancer category, variant category, and drug class for genome matched treatments (GMTs). **a** 154 GMTs FDA approved for the same tumor type. **b** 64 GMTs FDA approved for a different tumor type. See Table 2 for clinical trials. Each line represents one GMT. Some patients contribute multiple lines if they received multiple GMTs. Height represents the number of cases. Colors determined by variants. Other primary sites of cancer include unknown primary. Other variants identified include: FGF/FGFR path, MSI, VHL, ARID1A. Other drug classes include: ret inhibitor, idh inhibitor, smo inhibitor.

A total of 240 GMTs were identified in 206 patients. 178 patients (86%) received one GMT, 25 patients (12%) received two GMTs, and 3 patients (2%) received four GMTs. The most frequent GMT given was anti-PD1/PD-L1 inhibitors (*n* = 88; 37%), followed by PARP inhibitors (*n* = 35; 15%), anti-Her2 directed therapies (*n* = 32, 13%) and CDK4/6 inhibitors (*n* = 18, 8%) (Supplementary Table 2). 154 GMTs (64%) were FDA-approved for the tumor type (“on-label”); 64 GMTs (27%) were FDA-approved for a different tumor type (“off-label”), and 22 GMTs (9%) were received on clinical trials (Figs. 2 and Table 2). Of the patients treated on clinical trials, 6 GMTs were given through trials in Maine (4 of those either through the TAPUR or the NCI-MATCH trial, two through industry-sponsored trials). 16 of the clinical trial GMTs were given through clinical trials out of state.

**Table 2:** Genome matched treatments (GMT)

Drug Status	Cancer Category	Primary Cancer Site	Drug Class	Variant Class	Variants
FDA-approved tumor type (154)	Lung (33)	Lung	PD-1/L1 inhibitor (25)	MAPK pathway	KRAS (2)
				PD-L pos	PD-L1 expression (15); PD-L1 expression, TMB-H (1)
				TMB high	TMB-H (7)
			MAPK inhibitor (6)	MAPK pathway	KRAS (1); BRAF (4)
				other variant	MAP2K1 (1)
			EGFR inhibitor (2)	EGFR	EGFR (2)
	Breast (38)	Breast	Her2/neu inhibitor (15)	ERBB2	ERBB2 (15)
			CDK4/6 inhibitor (11)	Cell cycle reg	CDKN2A, ESR1 (1); CCND1, ESR1 (1); CCND1, PIK3CA (1); CCND1 (5)
				PI3K/AKT/MTOR pathway	ESR1, PIK3CA (1); PIK3CA (1)
				other variant	FGFR1 (1)
			PI3K/AKT/MTOR inhibitors (9)	PI3K/AKT/MTOR pathway	PIK3CA (8); RPS6KB1 (1)
			PD-1/L1 inhibitor (2)	PD-L pos	PD-L1 expression (2)
			PARP inhibitor (1)	other variant	PALB2 (1)
	Gynecologic (20)	Cervix	PD-1/L1 inhibitor (1)	PD-L pos	PD-L1 expression, TMB-H (1)
		Endometrium	PD-1/L1 inhibitor (5)	other variant	MSI: Instable, TMB-H (2)
				PD-L pos	PD-L1 expression, TMB-H (1); PD-L1 expression (1)
				TMB high	TMB-H (1)
		Fallopian Tubes	PARP inhibitor (8)	non BRCA DDR	BARD1 (1); NBN (1)
				BRCA	BRCA2 (2); BRCA1 (4)
		Ovaries	PARP inhibitor (6)	non BRCA DDR	ATM (2); ARID1A, PTEN (1)
				BRCA	BRCA1 (1)
				MAPK pathway	KRAS (2)
	Gastroesophageal (25)	Esophagus	PD-1/L1 inhibitor (12)	ERBB2	ERBB2 (1)
				other variant	MSI: Instable, PD-L1 expression, TMB-H (2)
				PD-L pos	PD-L1 expression, TMB-H (1); PD-L1 expression (8)
			Her2/neu inhibitor (7)	ERBB2	ERBB2 (7)
		Gastric	Multi-kinase inhibitor (2)	other variant	KIT (2)
			PD-1/L1 inhibitor (2)	PD-L pos	PD-L1 expression (2)
			Her2/neu inhibitor (1)	ERBB2	ERBB2 (1)
		Other	PD-1/L1 inhibitor (1)	other variant	MSI: Instable, PD-L1 expression, TMB-H (1)
	Urinary (12)	Bladder	PD-1/L1 inhibitor (3)	PD-L pos	PD-L1 expression, TMB-H (1); PD-L1 expression (2)
		Other	PD-1/L1 inhibitor (2)	PD-L pos	PD-L1 expression (2)
		Renal	Multi-kinase inhibitor (5)	other variant	VHL (5)
			PD-1/L1 inhibitor (2)	PD-L pos	PD-L1 expression (2)
	Colon (7)	Colon	PD-1/L1 inhibitor (6)	other variant	MSI: Instable, PD-L1 expression, TMB-H (1); MSI: Instable (1)
				PD-L pos	PD-L1 expression (2)
				TMB high	TMB-H (2)
			EGFR inhibitor (1)	EGFR	EGFR (1)
	Liver/biliary (2)	Liver	PD-1/L1 inhibitor (2)	PD-L pos	PD-L1 expression (2)
	Melanoma (10)	Melanoma, mucous membranes (GI and other)	PD-1/L1 inhibitor (1)	MAPK pathway	BRAF (1)
		Melanoma, ocular	PD-1/L1 inhibitor (1)	PD-L pos	PD-L1 expression (1)
		Melanoma, skin	MAPK inhibitor (4)	MAPK pathway	BRAF (4)
			PD-1/L1 inhibitor (3)	PD-L pos	PD-L1 expression (2)
				TMB high	TMB-H (1)
		Other	PD-1/L1 inhibitor (1)	MAPK pathway	BRAF (1)
	Pancreas (2)	Pancreas	PARP inhibitor (2)	BRCA	BRCA2 (2)
	Prostate (3)	Prostate	PARP inhibitor (3)	non BRCA DDR	ARID1A (1)
				BRCA	BRCA2, RAD51C (1); BRCA2 (1)
	Other (2)	Other	PD-1/L1 inhibitor (2)	PD-L pos	PD-L1 expression (2)
FDA-approved different tumor type (64)	Lung (7)	Lung	Multi-kinase inhibitor (3)	other variant	MET (1); FGFR1 (1); FGFR2 (1)
			PARP inhibitor (1)	non BRCA DDR	FANCI (1)
			PI3K/AKT/MTOR inhibitors (1)	PI3K/AKT/MTOR pathway	PTEN (1)
		Mesothelioma, Lung	PD-1/L1 inhibitor (1)	PD-L pos	PD-L1 expression (1)
			PI3K/AKT/MTOR inhibitors (1)	PI3K/AKT/MTOR pathway	PIK3CA (1)
	Breast (1)	Breast	Multi-kinase inhibitor (1)	other variant	KIT (1)
	Gynecologic (12)	Cervix	PARP inhibitor (1)	non BRCA DDR	FANCM (1)
		Endometrium	PARP inhibitor (4)	BRCA	BRCA2 (2)
				non BRCA DDR	RAD50 (1); ARID1A (1)
			Her2/neu inhibitor (1)	ERBB2	ERBB2 (1)
		Fallopian Tubes	CDK4/6 inhibitor (1)	Cell cycle reg	CCNE1 (1)
			Her2/neu inhibitor (1)	ERBB2	ERBB2 (1)
		Other	Multi-kinase inhibitor (1)	other variant	FGF23, FGF6 (1)
		Ovaries	CDK4/6 inhibitor (1)	Cell cycle reg	CDKN2A (1)
			FGFR inhibitor (1)	other variant	FGFR1 (1)
		Uterus	PARP inhibitor (1)	non BRCA DDR	FANCM, KMT2D (1)
	Gastroesophageal (2)	Esophagus	PI3K/AKT/MTOR inhibitors (1)	PI3K/AKT/MTOR pathway	STK11 (1)
		Gastric	EGFR inhibitor (1)	EGFR	EGFR (1)
	Brain (16)	Brain	PD-1/L1 inhibitor (6)	other variant	IDH1 (2)
				PD-L pos	PD-L1 expression (4)
			CDK4/6 inhibitor (3)	Cell cycle reg	CDK4, PIK3CB (1); CDKN2A (1); CDK4 (1)
			EGFR inhibitor (3)	EGFR	EGFR (3)
			PARP inhibitor (2)	BRCA	BRCA2 (1)
				non BRCA DDR	CHEK2 (1)
			Her2/neu inhibitor (1)	EGFR	EGFR (1)
			Multi-kinase inhibitor (1)	other variant	PDGFRA (1)
	Urinary (5)	Bladder	PARP inhibitor (1)	MAPK pathway	ATR, NRAS, PIK3CA, WRN (1)
			PI3K/AKT/MTOR inhibitors (1)	PI3K/AKT/MTOR pathway	TSC1 (1)
		Other	PD-1/L1 inhibitor (1)	PD-L pos	PD-L1 expression (1)
			other drug (1)	other variant	PTCH1 (1)
		Renal	FGFR inhibitor (1)	other variant	FGFR3 (1)
	Colon (4)	Colon	PARP inhibitor (2)	BRCA	BRCA2 (1)
				MAPK pathway	NRAS (1)
			MAPK inhibitor (1)	MAPK pathway	BRAF (1)
			PD-1/L1 inhibitor (1)	MAPK pathway	KRAS (1)
	Liver/biliary (5)	Bile Duct	FGFR inhibitor (2)	other variant	FGFR3 (1); FGFR2 (1)
			other drug (1)	other variant	IDH2 (1)
		Gall Bladder	Multi-kinase inhibitor (1)	other variant	KDR (1)
		Other	Multi-kinase inhibitor (1)	other variant	FGF6 (1)
	Pancreas (5)	Pancreas	PD-1/L1 inhibitor (3)	PD-L pos	PD-L1 expression (3)
			PARP inhibitor (1)	non BRCA DDR	ATM (1)
			MAPK inhibitor (1)	MAPK pathway	KRAS (1)
	Prostate (1)	Prostate	PD-1/L1 inhibitor (1)	PD-L pos	PD-L1 expression (1)
	Other (6)	Other	PD-1/L1 inhibitor (2)	PD-L pos	PD-L1 expression (2)
			MAPK inhibitor (1)	MAPK pathway	BRAF (1)
			CDK4/6 inhibitor (1)	Cell cycle reg	CDK4 (1)
		Unknown	PARP inhibitor (1)	Cell cycle reg	CDK12 (1)
			PD-1/L1 inhibitor (1)	PD-L pos	PD-L1 expression (1)
Clinical trial (22)	Lung (3)	Lung	MAPK inhibitor (3)	MAPK pathway	KRAS (3)
	Breast (3)	Breast	Her2/neu inhibitor (2)	ERBB2	ERBB2 (2)
			PARP inhibitor (1)	non BRCA DDR	ARID1A (1)
	Gynecologic (2)	Endometrium	PI3K/AKT/MTOR inhibitors (1)	PI3K/AKT/MTOR pathway	AKT1 (1)
			MAPK inhibitor (1)	MAPK pathway	BRAF (1)
	Gastroesophageal (1)	Gastric	Multi-kinase inhibitor (1)	other variant	KIT (1)
	Brain (4)	Brain	EGFR inhibitor (2)	EGFR	EGFR (2)
			CDK4/6 inhibitor (1)	Cell cycle reg	CDKN2A (1)
			MAPK inhibitor (1)	MAPK pathway	NF1 (1)
	Colon (4)	Colon	Her2/neu inhibitor (2)	ERBB2	ERBB2 (2)
			MAPK inhibitor (2)	MAPK pathway	NRAS (2)
	Liver/biliary (3)	Bile Duct	Her2/neu inhibitor (2)	ERBB2	ERBB2 (2)
			FGFR inhibitor (1)	other variant	FGFR2 (1)
	Pancreas (1)	Pancreas	PD-1/L1 inhibitor (1)	PD-L pos	PD-L1 expression (1)
	Other (1)	Other	other drug (1)	other variant	RET (1)

The matching process is described in detail in the supplementary materials, but briefly, GMT was defined as either: (a) patient received a drug with an FDA label that included a variant or biomarker identified by their GTT; and/or (b) a patient received a targeted drug that was listed on their GTT report. GMTs were further categorized into “FDA-approved within tumor type (on-label)”, “FDA-approved in different tumor type (off-label)” or “Clinical trial”. There were 240 GMTs among the 206 patients. N of cases is displayed in ().

In the “FDA-approved for the tumor type” category, breast cancer was the most common cancer category (*n* = 38, 25%) with Her2/neu inhibitors being the most common treatment (*n* = 15, 39%), followed by lung cancer (*n* = 33, 21%) treated with PD-1/PD-L1 inhibitors (*n* = 25, 76%) (Table 2). In the “FDA-approved different tumor type” category, brain tumors were the most common cancer category (*n* = 16, 25.0%) with PD-1/PD-L1 inhibitors (*n* = 6, 38%), CDK4/6 (*n* = 3, 19%), and EGFR (*n* = 3, 19%) inhibitors the most common treatments. Brain (*n* = 4, 18%) and Colon (*n* = 4, 18%) cancers were the most common tumor types within the “Clinical trial” category of GMTs (Table 2).

### Survival

**Fig. 3:**
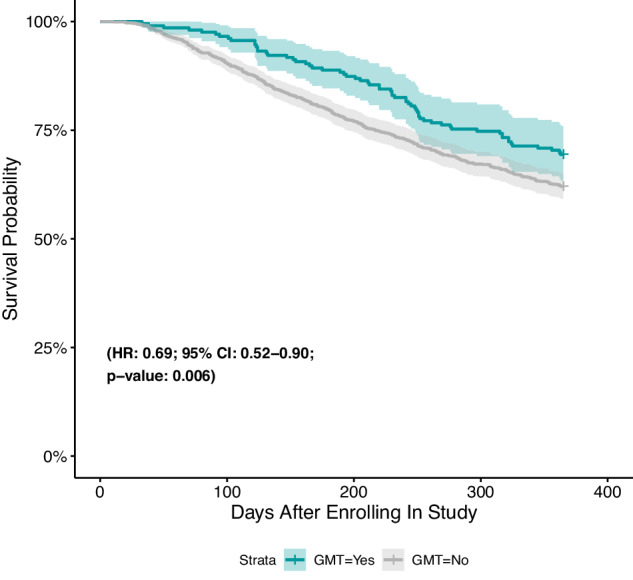
12-month overall survival. Kaplan–Meier survival curve displays crude, unadjusted survival. Hazard ratio using Inverse Probability of Treatment Weighting adjusts for age, sex, education, cancer stage, cancer site, and quality of life. Colored error bars represent 95% confidence intervals.

63 of the 206 patients (30.6%) in the GMT group died within 365 days of consent, compared to 399 of the 1052 (37.9%) in the non-GMT group. Using Inverse Probability of Treatment Weighting to adjust for imbalance in baseline characteristics, a Cox proportional hazards model demonstrated that patients who received GMT were 31% less likely to die within 1 year than those who did not receive GMT ((Fig. 3) HR: 0.69; 95% CI: 0.52–0.90; *p*-value: 0.006). Similar findings were obtained using univariate and multivariate models without Inverse Probability of Treatment Weighting (see supplementary materials).

## Discussion

This study evaluated the outcomes of patients enrolled in the Maine Cancer Genomics Initiative (MCGI), a community oncology initiative conducted between 2017 and 2020 in one of the most rural states in the US. We found that clinicians enrolled patients with diverse types of cancer but predominantly stage IV/metastatic disease. Genomic tumor test (GTT) reports identified “potentially actionable” variants and biomarkers in nearly all successfully tested patients, and 16% of patients went on to receive genome matched treatment (GMT). Although this initiative was not a randomized controlled trial designed to test the efficacy of specific drugs or GTT, we observed that patients who received GMT had increased overall survival at 12-months compared to patients who did not receive GMT.

The MCGI allowed patients to enroll with broad inclusion criteria. As expected, clinicians tended to enroll higher-stage patients (74% were Stage IV or Grade 4), but 26% were earlier stage patients for whom genome-matched treatment is rarely indicated. This pattern reflects the variety of ways GTT was used by clinicians in the MCGI: most tested only advanced cancer patients requiring a new therapy, but some tested early to have information available if conventional treatments failed. There was also diversity in the primary sites of cancer, demonstrating an interesting patterns of utilization. The high proportion of enrolled patients with primary brain and gynecologic tumors shows the high interest in GTT by specific clinicians—most gynecologic and neuro oncology patients receive care by a small number of sub-specialized oncologists who were highly motivated to offer participation in the study to their patients.

As previously reported^30^, the GTT failure rate in our study decreased over time and averaged out at approximately 14%. Both metrics are consistent with reports from other testing initiatives^31^. We found that most patients (97.5%) had at least one potentially actionable variant, which is higher than typically reported GTT programs^10^. This difference can be explained by different definitions of actionability—in our case, genomic variants with diagnostic, prognostic and therapeutic implications were defined as actionable on GTT reports whereas test reports in other programs may have focused on therapeutically relevant variants alone.

Consistent with recently reported outcomes of over 18,000 patients within the Veterans Administration (VA) system^23^, we found that ~16% of patients went on to receive GMT. This rate of administering GMTs was similar to other observational studies^10^, and reflects the challenges of administering GMTs. For some patients, other standard-of-care options may have been more appropriate than GMT, for other patients, GMTs may have been appropriate but not available. Not surprisingly, the rate of patients matched to targeted therapies is significantly higher in programs that include a study-driven matching algorithm such as the DRUP study in the Netherlands^9^ (46% of patients matched to a clinical trial) or the I-PREDICT study at University of California at San Diego^4^ (49% of patients received personalized treatment). While there are many inherent differences between study populations and setting (community in our case versus academic in others), one key feature of both DRUP and I-PREDICT was the systematic review of each case by a molecular/genomic tumor board, which made specific recommendations for each case. As described previously^30^, we also implemented a genomic tumor board program, however, case review focused on those cases that the physicians required specific input on rather than all cases. More details on the genomic tumor board will be forthcoming in a separate manuscript.

Overall, the most commonly received GMTs were either immunotherapy drugs (anti-PD-1/PD-L1), anti-Her2 targeting therapies, CDK4/6 inhibitors and PARP inhibitors. This distribution is expected given the high number of lung, breast and gynecologic cancer patients enrolled in the MCGI, which are often treated with these drugs. The distribution of treatments in our population is different compared to the VA population, likely due to a different distribution of tumor types (e.g., higher proportion of breast cancer and gynecologic cancer patients in our study population) and a higher proportion of male patients in the VA study.

To gain a better understanding of how patients accessed GMTs, we divided the GMTs into three categories. Almost two-thirds of GMTs (64%) were administered in a tumor type with an FDA-approved targeted therapy. This is not surprising given the strong representation of breast and lung cancer patients in our study, which have the most GMT options of all solid tumors^32^. Similarly, the most common GMT in either one of these groups (ERBB2/Her2-neu directed therapy in breast cancer; PD-1/PD-L1-targeted therapy in lung cancer) are commonly indicated and effective treatments for these two cancers. PARP inhibitors were well represented in the ovarian/fallopian tube cancer cohorts and less commonly observed in prostate and pancreatic cancer patients, likely because of narrower clinical indications in the latter two than the former.

Five percent of analyzed patients received a GMT in the “FDA-approved in other tumor type” (i.e., “off-label”) group. This is markedly higher than in the VA study, in which only 0.9% of patients received an “off-label” GMT^23^. This difference may be primarily a consequence of different drug access mechanisms between the VA and the hospitals represented in our study or differences in the demographics between the two study populations. Finally, only 1.7% of all patients in our study accessed GMT through a clinical trial, almost 75% of them through a clinical trial out of state at the closest academic medical centers with phase I/II trial programs. Even though both the MATCH and—towards the end of the study period—the TAPUR trials were open in Maine, they enrolled only 4 patients tested through the MCGI. This points to a larger access issue: in order to make GMTs available to a large group of cancer patients, access to GTT alone is not sufficient—access to treatments, ideally through clinical trials is also needed. The importance of clinical trials in the delivery of precision oncology is supported by a recent ASCO Provisional Opinion for somatic genomic testing^33^ and another recommendation from Australia^32^, which both prioritize clinical trial access over off-label drug use. Accordingly, a comprehensive clinical trial program with novel therapeutics should be made available geographically close to the patient’s home, as suggested previously^34^.

Interestingly, primary brain tumor patients constituted the largest group of patients receiving a GMT in the “off-label” setting. This may reflect the lack of effective conventional treatment options especially for patients with glioblastoma multiforme (GBM), with limited standard options available beyond upfront chemoradiation and adjuvant temozolomide^35^. Twelve percent of all tested GBM patients received a GMT in the off-label setting, and 3% through a genomically-matched clinical trial. These numbers are significantly different from a recent publication, in which 56% of IDH-wildtype GBM patients with GTT enrolled on a clinical trial while only 0.7% of patients received GTT-informed off-label therapy^36^. Again, this points to significant differences in access to clinical trials between urban centers and a rural state. Additionally, some of this may also be driven by clinicians’ practice preferences.

Patients who received and did not receive GMT were similar in sociodemographic characteristics such as income, education, and rurality. In the US, these social determinants of health typically create disparities in access to cancer care services^37^. The lack of such disparities in the receipt of GMT in our study suggests that features of the MCGI, such as free testing, state-wide reach, and genomic tumor boards, may have helped overcome inequities in access to treatment that might otherwise have limited access to GMT for vulnerable populations.

Though this project was not a randomized controlled trial designed to test the efficacy of GMT, we observed that those who received GMT had greater overall survival up to 12-months compared to those who did not receive GMT, even when adjusting for the propensity for individual patients to receive GMT. This finding needs to be interpreted with caution, given that the observational design of this study may still lead to bias due to unmeasured confounders. Despite these caveats, the overall findings suggest a potential survival benefit from GMT in community oncology settings and supports the value of further research to evaluate this possibility.

This study had several other unique characteristics that call for caution in interpreting the results. One of the main barriers of precision oncology implementation, cost of testing^38^, was removed in the MCGI, which likely increased the identification of potentially targetable genomic alterations and may have increased the prescription of GMT by physicians. In addition, GTT utilization by clinicians was not prescribed by the study; some clinicians used it early to have information for later, while others used it late—as a tool of last resort—and some used it primarily to further scientific research. This led to a heterogeneous study population with respect to cancer site and stage. In line with the population characteristics in Maine, the study population was primarily white and non-Hispanic, which limits the generalizability to populations with diverse racial and ethnic backgrounds. On the other hand, this study focused on a typically under-represented study population: cancer patients treated by community oncologists in a rural state. Furthermore, although the sample was limited to a single state, all oncology practices in the state participated and the sample was therefore regionally representative.

Another point to consider is the method used to determine GMT. Some matches were based on an association with strong evidence linking a biomarker to a drug (for example: ERBB2 amplification and anti-Her2 therapy). However, other matches from GTT reports were based on associations with relatively weak evidence (e.g., biologic rationale or clinical trial inclusion criteria; for example CCND1 amplification associated with CDK4/6 inhibitors). Especially in this latter example, we don’t know how much the GTT result actually influenced the treating physician’s decision making. However, we feel that it’s important to include scenarios like this in this analysis. Broadly, this reflects a challenge in the field more generally: while multiple guidelines have been developed for reporting and interpreting clinically relevant genomics data (e.g., CAP/ASCO/AMP guidelines^39^, ESCAT scale^40^, TOPOGRAPH^32^), there is no standard method of determining whether a patient received treatment informed or matched to their genomic test result—particularly in broad initiatives that enroll patients with numerous sites of cancer. Many existing research studies do not report explicit criteria for determining GMT (e.g., ‘Sequencing-directed therapy’)^1^. We believe our attempt to establish and transparently report such criteria represents a major strength of this study, but acknowledge that more work is needed to develop consensus on how to ascertain GMT.

There are a number of caveats with respect to the reported overall survival benefit of GMT compared to non-GMT at 12 months. We are planning an additional report with more mature follow-up in the future. Furthermore, the favorable survival of the GMT group may be influenced by the outcomes of patients who received PD-1/PD-L1 inhibitors, which have generally been associated with improved survival in many cancer types. Interestingly, an exploratory sub-analysis showed that the most pronounced difference in survival was observed between the patient group that received a GMT on a clinical trial versus the non-GMT group (Supplementary Fig. 2). While this may suggest that patients will do better if enrolled on a GMT-derived clinical trial, this survival difference is more likely a reflection of selection bias for fitter patients enrolling on a clinical trial. Lastly, the primary analysis in this study compared patients who receive at least one GMT to those that did not receive GMT. Since a recent study by Kato et al.^4^, demonstrated that patients who received more than one MTB-recommended treatment have better survival outcomes than those that only received one, it would be interesting to explore differences in overall survival between those groups. However, only a small number of patients received more than one GMT (*n* = 28), making a subgroup analysis difficult in our dataset.

Finally, in this paper, we focused on evaluating whether patients received treatment matched to their GTT results, but a larger question for the field is understanding whether testing has other effects on clinician and patient decision making or other outcomes. For instance, testing can rule out potentially ineffective treatments or otherwise help clinicians and patients decide on palliative vs. curative treatment goals. It can also have the psychological benefit—for both patients and clinicians—of fostering peace of mind that “no stones were left unturned.” Ascertaining these and other outcomes requires different data sources that are typically not captured in medical records.

In conclusion, this study reports the treatment outcomes of a statewide community-based precision oncology initiative that aimed to broadly disseminate and implement GTT in rural cancer care settings. We found that this initiative resulted in levels of GMT that were similar to other initiatives in larger academic settings, although a relatively larger fraction received off-label treatments. Furthermore, although this was an observational study that was not designed to test a survival difference, it yielded promising evidence for a potential 12-month survival benefit of patients receiving GMT. Taken together, these findings suggest that when disseminated and implemented with a supportive infrastructure, GTT may benefit cancer patients in rural community oncology settings. To fully realize the potential of genomically-driven oncology and provide more patients with genome-matched treatments, future programs will need to establish more effective clinical trial and treatment navigation infrastructure.

## Methods

### Study design and population

The MCGI was designed as an observational study that collected patient outcome data and provided access to GTTs, genomic education to clinicians, and clinical decision support through genomic tumor boards (previously described)^30^. Clinicians were invited to participate in the MCGI via site visits, telephone, and personal contact. Once enrolled, Maine oncologists (including medical, gynecologic and neuro-oncologists) were able to enroll patients to the MCGI study. The protocol allowed patient participants with any stage (any WHO grade for primary brain cancers), any solid malignancy and treatment, adequate functional status (Eastern Cooperative Oncology Group performance status 0–2) to enroll. Enrolled patients received a free tissue-based GTT using FFPE tissue and agreed to complete periodic surveys and data abstraction from their medical record. This paper analyzes data from patient participants who enrolled in MCGI between July 2017 and October 2020. We have previously reported the implementation of the MCGI and a variety of psychosocial outcomes^41–46^.

All patient participants were enrolled onto the study after an informed consent conversation with a trained research professional and provided written informed consent. All study-enrolled patient participants were given the opportunity to “opt-in” to the MCGI registry study, which makes the study data available for future, cancer-related research. The study was approved by New England IRB (NEIRB)/Western IRB (WIRB), all part of WCG. All research-involved participating institutions entered a reliance agreement with WCG. The study design and conduct complied with all relevant ethical regulations including the Declaration of Helsinki.

### Genomic tumor tests

Between July 2017 and March 2019, 346 samples were analyzed using the ActionSeq Plus from the JAX Clinical Genomics Laboratory. This test consisted of the DNA-based JAX ActionSeq™ assay analyzing single nucleotide variants (SNVs), insertion/deletions (indels) and copy number variants (CNVs) in 212 cancer-related gene exons, and the RNA-based JAX FusionSeq™ (ArcherDx), detecting fusions involving one or more of 53 genes known to be associated with various carcinomas, sarcomas and hematologic malignancies.

Between June 2018 and September 2019, 416 samples were analyzed on the TruSight Tumor 170 (TST-170, Illumina Inc.) NGS platform available through a partnership with Navican (Navican TheraMap™). This panel evaluated the DNA of 156 cancer-related gene exons for SNV’s, indels, CNV’s and the RNA of 54 cancer-related genes for fusions. The panel also reported out tumor mutation burden (TMB) based on 500 kb of sequenced DNA and Microsatellite Instability (MSI) status based on a standard PCR. PD-L1 testing using the SP263 IHC assay was available at the request of the ordering physician.

Between April 2019 and December 2020, 840 samples were tested on the ActionSeq™ 2.0 Plus, which incorporates a DNA‐based panel (ActionSeq™ 2.0) comprising 501 cancer related genes for which all coding exons are sequenced and clinically significant variants in 209 genes are reported, and a RNA‐seq based panel (FusionSeq™ 2.0) evaluating the transcriptome for 548 genes known to form fusions in solid tumors and reporting clinically significant fusions across 53 gene partners. Tumor mutation burden (TMB) was calculated as the mutations per megabase (mut/Mb) across the ~2.3 Mb of coding DNA captured by the ActionSeq™ 2.0 panel. MSI status was evaluated based on the number of measured frameshift mutations per Mb of DNA. PD-L1 testing was also available at the request of the ordering clinician. All of the MCGI-associated clinical tests were delivered in a comprehensive test report. The reports identified potentially actionable tumor variants and biomarkers (based on previous guidelines)^39^, along with FDA approved drugs or experimental drugs through clinical trials to target those variants (for additional details see previous publication)^30^.

Variants and biomarkers in this analysis identified in tumors were abstracted from the GTT test reports; actionable gene variants and I/O markers and associated potential therapies (as identified by the testing laboratory) were entered into the matching process described below.

### Genome-matched treatment

To understand whether the GTTs were associated with patients receiving targeted treatment, we analyzed all treatments administered to patients after GTT results were returned and within 1 year of enrollment. Genomically matched treatment (GMT) was defined if a drug matched either of two criteria.

1. Drug-biomarker match: this criterion was met if a patient received a drug with an FDA label that included a variant or biomarker identified on their GTT report (either in the specific tumor type or another tumor type). For example, a patient with an ERBB2 mutation/amplification receiving an anti-her2 monoclonal antibody (e.g., Trastuzumab or Pertuzumab) in any tumor type was included in this group. Since PD-L1 results were included on most of the GTTs, we included PD-L1 + /PD-1 inhibitor as an acceptable drug-biomarker match in this match.
2. Drug-GTT report match: this criterion was met if a patient received a targeted drug that was listed on their GTT report as associated with a genomic variant, based on FDA approval status, existing evidence or based on clinical trial inclusion criteria. For example, a patient with a CDKN2A deletion receiving a CDK4/6 inhibitor (e.g., Palbociclib or Abemaciclib) was included in this group.

Full details for determining GMT are described in the supplementary materials. Patients could receive more than one GMT over the course of their treatment.

GMTs were then categorized into one of three possible groups: (1) The GMT was FDA approved in the same tumor type, i.e., “on-label” based on the drug-tumor type match (e.g., trastuzumab in ERBB2-amplified/Her2-overexpressing breast cancer); (2) The GMT was FDA approved but not in the patient’s tumor type, i.e., “off-label” (e.g., Olaparib in BRCA1-mutated primary brain tumor); or (3) treatments were administered through a clinical trial with genomic marker-derived inclusion criteria. Of note, we included targeted therapies that are FDA-approved in a specific tumor type in the “FDA-approved in the same tumor type” even if the FDA label did not have an associated biomarker as these drugs are often associated with a biologically relevant biomarker on the test report. For example, CDK4/6 inhibitors in hormone receptor positive (HR+) breast cancer are FDA-approved in a biomarker-independent fashion, yet many HR+ breast cancer cases exhibit CCND1 amplification, which is biologically linked to CDK4/6 inhibitors and therefore identified on genomic test reports. Since we cannot exclude that test reports linking CCND1 amplifications with CDK4/6 inhibitors influenced physicians’ decision-making, we included CDK4/6 inhibitors in breast cancer as GMT in the “FDA-approved in the same tumor type” group. Furthermore, treatments in a tumor type that was included in the FDA label were included in the “FDA-approved in the same tumor type” category even if the patient only had a biomarker that was not included on the FDA package insert, e.g., Olaparib in an ovarian cancer with a FANCA mutation.

### Sociodemographic and clinical variables

Sociodemographic variables (age, gender, race/ethnicity, income, rurality) and quality of life^47^ were reported by patients in a survey. Patients completed surveys within 14 days of enrollment either online through the REDCap Cloud® data platform, or by paper with responses entered by research coordinators. Clinical cancer diagnoses were grouped by the following categories: lung, breast, gynecologic, gastroesophageal, brain, urinary, colon, liver/biliary, melanoma, pancreas, prostate, gynecologic, brain, and “other”. See supplementary materials for more details.

### Mortality

Date of death for each patient was ascertained up to 12 months after enrollment. Site research coordinators reviewed each patient’s medical records and if there was no indication of the patient being alive at end of study, they reviewed public sources (i.e., obituaries) to identify possible date of death. Patients without documented death at 1 year were treated as alive.

### Data analysis

Descriptive statistics were calculated for demographic variables, primary cancer sites and stage, stratified by GMT status (Table 1). We determined the results of GTTs in terms of variants and biomarkers identified, and examined whether the GTT results matched treatments as described above (i.e., GMT). To explore how receiving GMT was associated with mortality, survival outcomes were assessed using Kaplan–Meier survival curves and Cox proportional hazard regression analysis. Cox proportional hazards regression analysis was conducted with mortality as the outcome variable (enrollment to maximum of 12 months of follow-up), and GMT status as the independent variable.

To address potential selection bias in our non-randomized study, we used Inverse Probability of Treatment Weighting, which allowed us to balance the two groups of patients: those that received GMT and those that did not. First, we calculated the probability (propensity) of patients receiving GMT, given their individual characteristics. Second, weights were determined for each individual as the inverse of the probability of receiving GMT. These weights created a dataset in which measured confounders were equally distributed across two groups. As previously described^48,49^, we used only covariates that were statistically related to exposure and outcome, or outcome alone, but not related only to exposure. Using simple univariate regressions of each covariate against treatment and outcome, we identified age, sex, education, cancer stage, cancer site, and quality of life as covariates for weighting, and rejected ethnicity, household income and rurality.

### Reporting summary

Further information on research design is available in the Nature Research Reporting Summary linked to this article.

### Supplementary information

Supplementary Material

REPORTING SUMMARY

## Data Availability

At the request of the corresponding author, de-identified data are available for any requests related to the content and analyses presented in this manuscript. Data from the patient participants that opted-in to have their data included in the the MCGI registry (>95% of all patient participants in this manuscript) are available for future cancer-related research through the MCGI Registry. Qualified researchers can apply for access to the datasets via the MCGI Registry by contacting mcgi@jax.org. If a request is approved, the datasets will be made available via data use agreements with The Jackson Laboratory.

## Code availability

The analytic code that generated the findings of this study are available through an online repository (https://osf.io/5fhkg/?view_only=1d52786ff08f46f5bb9c546c5c76a366). All analyses were performed using R version 4.1.2^50^.
