# Supplementary material for: Genome-matched treatments and patient outcomes in the Maine Cancer Genomics Initiative (MCGI)
Source: NPJ Precis Oncol. 2024 Mar 9;8:67. doi: 10.1038/s41698-024-00547-4 (PMC10924947; doi:10.1038/s41698-024-00547-4)
Supplement: Supplementary file 1 — Supplementary Material [file 41698_2024_547_MOESM1_ESM.pdf]

Anderson et al., Genome-matched treatments and patient outcomes in the Maine Cancer Genomics Initiative (MCGI), a prospective, observational study in a rural state

Supplemental Information

Table of contents in supplemental materials

S1. Verbose description of genome matched treatment

S2. Additional details about sociodemographic variables

S3. Additional figures and tables

Supplemental Table 1: Complete list of variants identified by testing

Supplemental Table 2: Complete list of genome matched treatments by identification method

Supplemental Figure 1: Kaplan Meier curve stratified by site of cancer

Supplemental Figure 2: Survival stratified by categories of GMT

S4. Additional statistical models of survival

S5. Example of genomic tumor test report

S6. Supplemental references

S7. Supplementary Note

S1. Detailed description of genome matched treatment

Below is the detailed description of the process of determining whether each participant received genome matched treatment (GMT) in the Maine Cancer Genomics Initiative. A condensed version appears in the main paper, with the verbose version being presented here in the supplemental materials.

To understand how the genomic tumor tests (GTTs) offered through the Maine Cancer Genomics Initiative (MCGI) led to targeted treatment, we determined whether each patient received a treatment that was matched to results found on their MCGI GTT (henceforth genomically matched treatment, GMT).

After testing, a GTT test report was generated and returned to clinicians with potentially actionable biomarkers and variants listed (Tier 1 & Tier 2 variants; see example of test report in supplemental materials below). Tier 3, or variants of unknown significance, were categorized as non-actionable variants. Reports also included a curated list of FDA approved drugs and clinical trials to target those variants identified. These categorizations and recommendations were made by The Jackson Lab CLIA staff using the JAX Clinical Knowledgebase (JAX CKB; for details see[1)] and other publically available sources for the JAX test. The NAVICAN test reports used a similar system.

Starting with patients who had genomic tumor test results returned to their physicians, each participant’s genomic tumor test report was examined to determine whether any actionable biomarkers were identified by the test (i.e. Tier 1 & Tier 2 variants and I/O biomarkers). Tier 3, or

variants of unknown significance, were categorized as non-actionable variants and were not analyzed further.

Next, we examined patients' cancer treatment data, which was abstracted from electronic medical records by research site staff. For patients with potentially actionable variants, we examined the study records and created a list of all treatment drugs that all patients received starting after the test report date until 12-months after enrollment for each patient.

Next, two approaches were used to determine whether each patient received a GMT. Matching using these approaches was based on drug class (e.g. PARP inhibitor) rather than specific drug name (e.g. Olaparib).

A) First, matching criteria were identified by creating a list of pre-defined gene targets (based on FDA drug labels and clinical trial inclusion criteria for targeted drug; see "FDA label match" columns in Supplemental Table 2). If a patient received one of these drugs and had one of the identified biomarkers on their GTT report, they were classified as receiving GMT—even if the GTT report did not list the drug as a recommended treatment. This approach was used because some reports might have missed emerging targeted options that became available after the GTT report was issued. For example, if a patient had biomarker ERBB2, and received an anti-her2 MAB drug (such as Trastuzumab), that was considered a match because the FDA label for Trastuzumab includes ERBB2 biomarker as criteria for appropriate use. These matching criteria were created by JR and reviewed by MH. FDA labels change over time and were reviewed in July 2022, so not all of the MCGI clinicians were likely aware of all pairings during the years of the initiative (2017-2021).
B) Second, additional matches were identified when patients received drugs of the same class of drugs recommended on their GTT report (see Supplemental Table 2, "GTT report match" columns). For example, if the test report identified a patient as having an activating BRAF mutation, and suggested a PD-L1 inhibitor due to a clinical trial enrolling based on activating BRAF mutations, and the participant actually received a PD-L1 inhibitor, that participant was categorized as receiving GMT. We note that the evidence behind these recommendations is constantly fluctuating, but this approach captured recommendations at the time test reports were created.

S2. Additional details about sociodemographic variables

Education level was assessed using five response categories: "Less than high school," "High School Graduate/GED," "Some college/Trade School," "Bachelor's Degree" and "Graduate Degree (Masters, PhD, etc.)". For this analysis, the bachelor's degree and graduate degree levels were collapsed due to small numbers in each category. Household income was assessed by patients' self-reported household income categories. The rurality of each participant's primary residence was determined via the United States Department of Agriculture's Rural-Urban Commuting Area (RUCA) codes which uses measures of population density, urbanization, and daily commuting to map ZIP codes to urban/rural categories[2]. Because of small numbers of patients in some categories, we collapsed RUCA codes into 3 categories: 1 = "metro," 2-6 = "large rural," and 7-10 "small/isolated rural."

S3. Additional tables and figures

Supplemental Table 1: Complete list of variants identified by testing, across all patients enrolled

Variant	n
TP53	641
PD-L1 expression	318
KRAS	233
APC	166
PIK3CA	163
TMB-H	130
PTEN	109
ARID1A	108
CDKN2A	99
EGFR	84
ERBB2	71
NF1	51
FBXW7	43
ATM	42
BRAF	39
RB1	39
IDH1	38
FGFR1	36
CCNE1	35
KMT2D	34
MYC	33
CDK4	32
CCND1	31
MSI: Instable	31
PIK3R1	31
BRCA2	29
FGF19	27
CTNNB1	26
FGF3	26
MDM2	25
STK11	25
BRCA1	24
FGF4	21
NRAS	20
SMAD4	20
RNF43	19
MUTYH	18
VHL	17
AKT2	16
ESR1	16
KMT2C	16
AKT1	15
ATR	15
BAP1	15
TMPRSS2	15
CREBBP	13
PDGFRA	13
PTPN11	13
JAK2	12
NBN	12
ARID2	11
ATRX	11
CDH1	11
FANCM	11
JAK1	11
KIT	11
MET	11
NFE2L2	11
PTCH1	11
RAD50	11
SLX4	11
BRIP1	10
EP300	10
MSH3	10
MSH6	10
AR	9
CDK12	9
FGFR3	9
LRP1B	9
PALB2	9
PBRM1	9
CDKN1B	8
GLI1	8
KDM6A	8
KDR	8
MSH2	8
TSC2	8
CCND3	7
CHEK2	7
ERBB3	7
FGF6	7
HDAC2	7
KEAP1	7
MAP2K1	7
MTOR	7
MYCN	7
NOTCH1	7
RET	7
SMARCA4	7
CDK6	6
FANCA	6
FGF10	6
FGFR2	6
HRAS	6
LAMP1	6
MCL1	6
PAK1	6
TNK2	6
BLM	5
GNAS	5
MAPK1	5
MLH1	5
NF2	5
NOTCH3	5
RPS6KB1-VMP1	5
TSC1	5
WRN	5
AXIN1	4
BARD1	4
EGFR-SEPT14	4
FGF23	4
FLCN	4
IDH2	4
MDM4	4
MLH3	4
MYCL	4
PIM1	4
PMS2	4
RPS6KB1	4
SRC	4
TGFBR2	4
CRKL	3
EPHB4	3
FANCE	3
FANCL	3
FGFR4	3
GNA11	3
KMT2A	3
PIK3CB	3
PPM1D	3
RAD51C	3
RICTOR	3
TMPRSS2-ERG	3
CAPZA2-MET	2
CCND2	2
DDR2	2
ERCC2	2
ERG	2
ESR1-CCDC170	2
FANCF	2
FANCI	2
FGF9	2
FRK	2
IRS2	2
JUN	2
MEN1	2
MRE11	2
PIK3CD	2
POLB	2
SDHB	2
SMO	2
AGK-BRAF	1
ALK	1
ARID1	1
ATP6V0A1-ERBB2	1
BCOR	1
BCR	1
BRCA1-ACLY	1
CD274	1
CD74-NRG1	1
CDKN1A	1
CHOP	1
CIC	1
CTNINB1	1
DYRK1B	1
EIF3E-RSPO2	1
EML4	1
EPHA2	1
ERBB4	1
ERCC3	1
ESR1-PFN2	1
FAM175A	1
FANCC	1
FGF18	1
FGF7	1
FGFR2-BICC1	1
FGFR2-MYPN	1
FGFR3-TACC3	1
FGRF3	1
FOXL2	1
GNAQ	1
GTF2IRD1-MET	1
GU1	1
IGF1R	1
JAK3	1
KIAA1549-BRAF	1
KMTsD	1
MSH2-TSGA10	1
MST1R	1
NOTCH2	1
NRG1	1
NTRK3	1
PIK3R2	1
PMS1	1
PPP2R1A	1
PRBM1	1
QKI-BRAF	1
RAB11FIP1-NRG1	1
RAD51	1
RAD54L	1
RAF50	1
SAMARCA4	1
SEPT7-MET	1
SHANK2-PIK3CA	1
SMARCB1	1
SOX6-RAF1	1
SYK	1
TERT	1
TOP2B-RAF1	1
TP53-CEP104	1
VEGFA	1

Supplemental Table 2: Complete list of genome matched treatments by identification method

Drug class	Drug names	Biomarker, FDA gene match	n, gene matches	Biomarker, GTT drug match	n, drug matches	Total
akt inhibitor	ipatasertib	AKT1	1		0	1
anti-egfr adc	depatuxizumab mafodotin	EGFR	1		0	1
anti-egfr mab			0	EGFR	1	1
anti-her2 adc	trastuzumab emtansine, fam-trastuzumab deruxtecan-nxki	ERBB2	9		0	9
anti-her2 mab	trastuzumab, pertuzumab	ERBB2	18		0	18
anti-pd-1 mab	nivolumab, pembrolizumab	MSI: Instable, PD-L1 expression, TMB-H	75	BRAF, ERBB2, IDH1, KRAS	7	82
anti-pd-l1 mab	atezolizumab	PD-L1 expression	5	KRAS	1	6
cdk4/6 inhibitor			0	CCND1, CCNE1, CDK4, CDKN2A, ESR1, FGFR1, PIK3CA, PIK3CB	18	18
egfr inhibitor	afatinib, osimertinib	EGFR	7		0	7
fgfr inhibitor	tas-120, erdafitinib	FGFR2, FGFR3	4	FGFR1	1	5
her inhibitor	tucatinib, lapatinib, neratinib	ERBB2	4	EGFR	1	5
idh inhibitor	enasidenib mesylate	IDH2	1		0	1
kras g12c inhibitor	MRTX 849, sotorasib	KRAS	2		0	2
mek inhibitor	trametinib, binimetinib	BRAF	7	KRAS, MAP2K1, NF1, NRAS	6	13
mtor inhibitor			0	PTEN, RPS6KB1, STK11, TSC1	4	4
multikinase inhibitor	imatinib	KIT	2	FGF23, FGF6, FGFR1, FGFR2, KDR, KIT, MET, PDGFRA, VHL	14	16
parp inhibitor	olaparib, rucaparib, niraparib	ATM, BRCA1, BRCA2, CDK12, CHEK2, PALB2, RAD51C	21	ARID1A, ATR, BARD1, FANCI, FANCM, KMT2D, KRAS, NBN, NRAS, PIK3CA, PTEN, RAD50, WRN	14	35
pi3k inhibitor	alpelisib, copanlisib	PIK3CA	9		0	9
raf inhibitor	LXH254, dabrafenib, encorafenib	BRAF, KRAS	5		0	5
ret inhibitor	LOXO-292	RET	1		0	1
smo inhibitor			0	PTCH1	1	1

Supplemental Figure 1: Kaplan Meier curve stratified by site of cancer

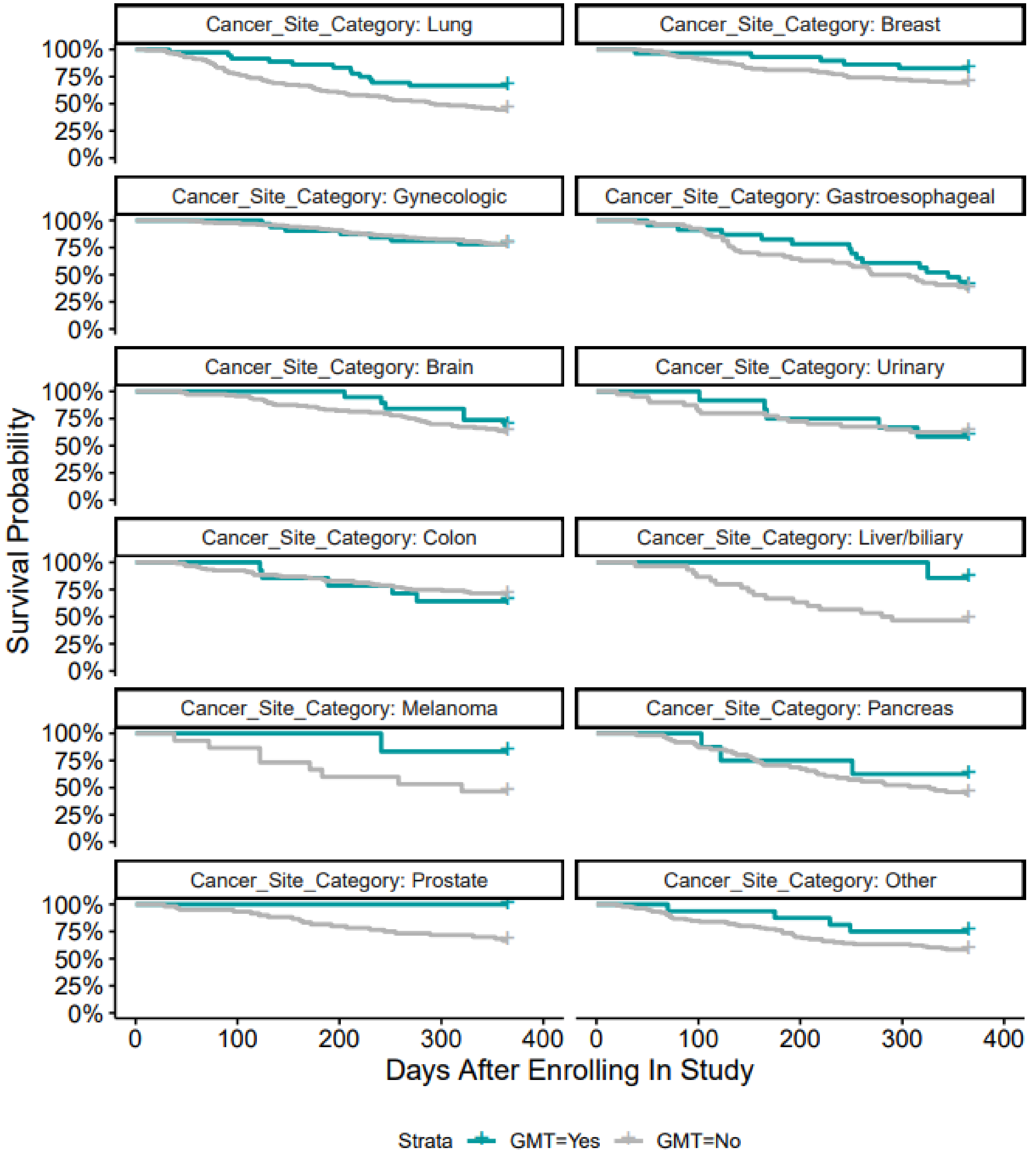

S4. Additional statistical models of survival

Additional models were run to understand the 12-month survival of patients. First, a univariate Cox proportional hazards model using only the treatment as a predictor showed a significantly better 12-

month survival (HR: 0.74; 95% CI: 0.57-0.96; p-value: 0.026) for the GMT group. Next a Cox model adjusting for potential confounders – age, sex, ethnicity, household income, education, rurality, cancer stage, cancer site, and quality of life – also showed significant results, with a lower hazard ratio for the GMT group (HR: 0.55; 95% CI: 0.40-0.75; p-value: 0.000). These findings are consistent with the analysis in the main manuscript using Inverse Probability of Treatment Weighting which found patients who received GMT were less likely to die.

We also conducted exploratory survival analyses looking at the different types of GMTs. We prepared Kaplan-Meier survival curves (Supplemental Figure 2) and simple, uncontrolled Cox proportional hazard models for the four categories of treatment: patients who received GMTs approved by the FDA for the same tumor type; GMTs FDA approved for a different tumor type, GMTs given through clinical trials, and patients who did not receive GMTs. In general, the analysis showed that patients with “GMTs FDA approved in tumor type” had a significant and moderately better survival (HR: 0.707; 95% CI: 0.519-0.962; p-value: 0.027) compared to patients who did not receive GMTs. Patients with GMTs “FDA approved for a different tumor type” had survival that was roughly the same as non-GMT patients (HR: 1.00; CI: 0.676-1.494; p-value: 0.980). Patients who received “GMTs through clinical trial” had a much higher survival (HR: 0.096; CI: 0.014-0.686; p-value: 0.020) compared to non-GMT patients. We note that these descriptions should not be interpreted causally. We do not believe it is likely that giving patients investigational GMTs through clinical trials increased their survival. Rather, heathier patients likely enrolled in clinical trials. Additionally, because some patients received multiple GMTs, these groups are not completely independent.

Supplemental Figure 2: Survival stratified by categories of GMT

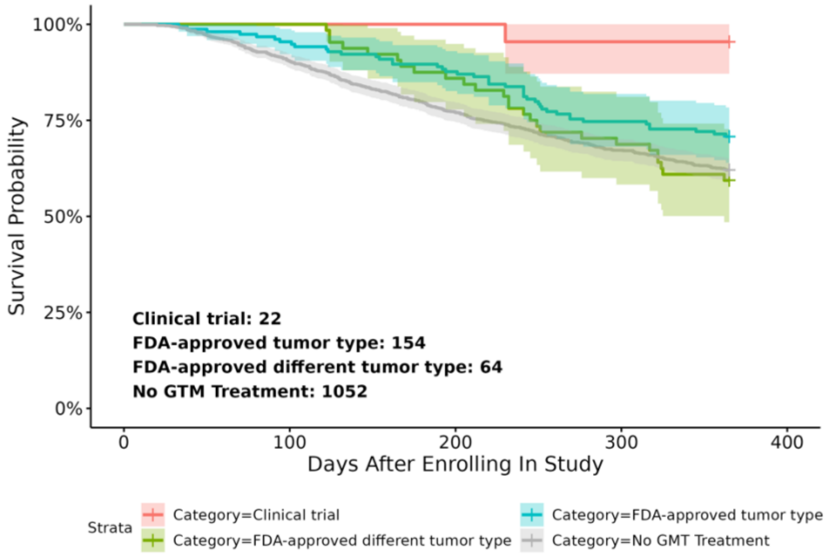

S5. Example of genomic tumor test report (Page 1 of 6)

The Jackson Laboratory

Patient Name:

Patient ID:

Test: ActionSeq™ 2.0 Plus

Report Date: 2019

PATIENT	SPECIMEN	PHYSICIAN
**Name:**	**Specimen ID:**	**Name:**
**Patient ID:**	**Source Specimen ID:**	**Affiliation:**
**Source Patient ID:** MCGI ID#1022	**1° Tumor Site:** N/A	
**D.O.B:**	**Specimen Site:** Left axilla lymph node	**Address:**
**Gender:** Female	**Specimen Type:** FFPE Slides	
**Tumor Type:** Metastatic breast cancer	**Neoplastic Content:** 90%	**Phone:**
	**Received Date:** 2019	**Collection Date:** 2019

Test Results Summary

[2] Clinically Significant Variants		Immunotherapy Markers	
[9] FDA Approved Drugs	[20] Clinical Trials	*TMB: 6.2 mut/Mb	MSI: Stable

This patient has one variant of strong clinical significance (Tier I) in the gene PIK3CA and one variant of potential clinical significance (Tier II) in the gene KRAS. No fusions of clinical significance were identified. Clinical correlation is REQUIRED.

*Tumors containing ≥20 mut/Mb are classified as TMB-High and may respond to immunotherapy treatment (PMID: 28835386).

Clinically Significant Variant(s)

Variants with Therapeutic Relevance

Genomic Marker	Class	Potential Therapies	FDA Approved–Tumor Type	FDA Approved - Different Indication	Potential Clinical Trials	Indicated Resistance
PIK3CA p.H1047R c.3140A>G	Tier I	✓ PI3K Inhibitor<br>✓ CDK4/6 Inhibitor<br>✓ mTOR Inhibitor<br>o AKT Inhibitor<br>o Androgen Synthesis Inhibitor	✓ Alpelisib<br>✓ Palbociclib	✓ Temsirolimus	9	NONE
KRAS p.G12A c.35G>C	Tier II	✓ Anti-PD-1 mAb<br>✓ MEK Inhibitor<br>✓ HER Inhibitor<br>✓ PARP Inhibitor<br>o WEE1 Inhibitor<br>o RAF Inhibitor<br>o ERK Inhibitor<br>o SHP2 Inhibitor<br>o TRK Inhibitor	NONE	✓ Avelumab<br>✓ Binimetinib<br>✓ Cobimetinib<br>✓ Neratinib<br>✓ Talazoparib<br>✓ Trametinib	12	NONE

✓ = Approved therapies, o = experimental therapies; Tier I = Strong clinical significance, Tier II = Potential clinical significance

Variants with Prognostic or Diagnostic Relevance

Genomic Marker	Class	Associated Tumor Type	Associated Clinical Outcome	Comments
NO VARIANTS OF PROGNOSTIC OR DIAGNOSTIC SIGNIFICANCE WERE IDENTIFIED				

✓ = Professional guidelines, o = Cohort studies; Tier I = Strong clinical significance, Tier II = Potential clinical significance, Px = Prognostic, Dx = Diagnostic

Note: Reporting of variants is done with priority given to therapeutic relevance. Variants that fit all or two of the three categories will only be reported in the category of the highest precedence; therapeutic>prognostic>diagnostic relevance. Therefore, these alterations may have unreported prognostic or diagnostic relevance to the patient. Potential therapies includes those drug classes with FDA approved drugs and/OR drugs in live clinical trials for genes or genomic alterations identified in the patient and/OR drugs with clinical evidence relevant to genes or genomic alterations identified in the patient.

Genomic Marker Interpretations

This patient has one variant of strong clinical significance (Tier I) in the gene PIK3CA and one variant of potential clinical significance (Tier II) in the gene KRAS. Mutations in PIK3CA and KRAS are detected in approximately 35% and 1% of breast cancer patients, respectively (PMID: 28481359). Clinical correlation is REQUIRED.

Gene	Variant	VAF/copy	Depth	Functional impact	Class	Type
PIK3CA	p.H1047R	34.0%	1188X	Gain of Function	Tier I	Therapeutic

PIK3CA H1047R is a single amino acid substitution in exon 21, which codes for part of PI3K kinase catalytic domain (InterPro: IPR000341). H1047R is a well characterized gain of function hotspot mutation resulting in constitutive activation of the PI3K/AKT/mTOR pathway (PMID: 24080956, PMID: 16432179, PMID: 15647370). This variant is found in a variety of cancers including cervical, colorectal, liver, gastric, lung, and bladder tumors (PMID: 24080956, PMID: 26567140, PMID: 28808038). This variant is absent or observed at extremely low frequency in large population cohorts and is present in the COSMIC database. Clinical correlation is REQUIRED.

S5. Example of genomic tumor test report (Page 2 of 6)

The Jackson Laboratory

Patient Name:
Patient ID:

Test: ActionSeq™ 2.0 Plus
Report Date: 2019

The PIK3CA gene encodes phosphatidylinositol 4,5-bisphosphate 3-kinase catalytic subunit alpha isoform (p110 alpha), a key regulator of cell proliferation, migration and survival via the PI3K/AKT pathway (PMID: 25688137, PMID: 16449998). The majority of oncogenic PIK3CA mutations are missense variants clustered in the helical and kinase domains, resulting in increased activation of the AKT/mTOR pathway. The most recurrently mutated hotspots are located at amino acid residues 542/545 in the helical domain, and 1047 in the kinase domain (PMID: 15016963, PMID: 25688137, PMID: 19075596). Gain of function PIK3CA mutations are well-established oncogenic events in a variety of cancers including colorectal, breast, liver and ovarian tumors (PMID: 16449998, PMID: 20535651). Alpelisib is FDA approved for hormone receptor (HR)-positive, human epidermal growth factor receptor 2 (HER2)-Negative, PIK3CA-mutated, advanced or metastatic breast cancer, and a number of approved and experimental drugs are being investigated in both clinical and preclinical studies. PIK3CA mutation status may have therapeutic, prognostic and/or diagnostic significance depending on the tumor type. Clinical correlation is REQUIRED.

KRAS	p.G12A	61.0%	788X	Gain of Function	Tier II	Therapeutic

KRAS G12A is a single amino acid substitution in exon 2, which codes for part of the P-loop containing nucleoside triphosphate hydrolase domain (InterPro: IPR027417). The G12A mutation is the least frequent of codon 12 alterations, but has been extensively characterized in the scientific literature (PMID: 20147967, PMID: 24132290, PMID: 21779504). Codon 12 mutants impair GTPase activity, leading to constitutive activation of downstream signaling (PMID: 23497191). Functional studies have shown G12A exhibits low levels of intrinsic GTPase activity and a high affinity for RAF, which may result in preferentially sustained RAF pathway activation and subsequent sensitivity to MEK inhibitors in cell lines (PMID: 26037647). G12A is a recurrent mutation found in colorectal and lung cancer and has also been detected in leukemia (PMID: 26662311, PMID: 26929424, PMID: 23092099). This variant is absent or observed at extremely low frequency in large population cohorts and is present in the COSMIC database. Clinical correlation is REQUIRED.

KRAS is a member of the small GTPase superfamily and a key regulator of the MAPK, PI3K/AKT/mTOR pathways (PMID: 23622131). Cohort studies have demonstrated that activating mutations resulting in oncogenic KRAS cluster into hotspots, most frequently at codons 12, 13 and 61 (PMID: 20147967, PMID: 24132290, PMID: 21779504). Variants in this gene are associated with a variety of cancers including pancreatic, colorectal and lung tumors (PMID: 26701267, PMID: 27010960). There are currently no FDA-approved drugs indicated for KRAS-mutant cancers, however approved and experimental drugs are being investigated in both clinical and preclinical studies. KRAS mutation status may have therapeutic, prognostic and/or diagnostic significance depending on the tumor type. Clinical correlation is REQUIRED.

VAF = Variant allelic fraction

FDA Approved Therapies – Patient Indication

Alpelisib — **PI3K Inhibitor** — ***PIK3CA***

Alpelisib (PIQRAY) is a phosphatidylinositol-3-kinase (PI3K) inhibitor, FDA approved in combination with fulvestrant for the treatment of postmenopausal women, and men, with hormone receptor (HR)-positive, human epidermal growth factor receptor 2 (HER2)-negative, PIK3CA-mutated, advanced or metastatic breast cancer as detected by an FDA-approved test following progression on or after an endocrine-based regimen (Alpelisib [package insert]. Novartis; 2019 [cited 2019 July]).

Palbociclib — **CDK4/6 Inhibitor** — ***PIK3CA***

The CDK4/6 inhibitor Palbociclib (Ibrance) is FDA-approved in combination with an aromatase inhibitor as an initial endocrine based therapy in postmenopausal women or in men, and in combination with fulvestrant in patients with disease progression following endocrine therapy, for the treatment of hormone receptor (HR)-positive, human epidermal growth factor receptor 2 (HER2)-negative advanced or metastatic breast cancer (Palbociclib [package insert]. Pfizerl 2019 [cited June 2019). Palbociclib is currently being investigated in clinical trials recruiting PIK3CA-altered tumors (clinicaltrials.gov).

FDA Approved Therapies – Different Indication

Avelumab — **Anti-PD-1 mAb** — ***KRAS***

Avelumab (Bavencio) is a monoclonal antibody binds to and blocks human immunosuppressive ligand programmed death-ligand 1 (PD-L1) protein signaling, resulting in immune regulation and antitumor immunity (PMID: 26014098). Avelumab is FDA-approved for use in patients 12 years and older with metastatic Merkel cell carcinoma, and in patients with locally advanced or metastatic urothelial carcinoma. Please see prescribing information for complete indication details (Bavencio [package insert], Pfizer; accessed May 2019). Avelumab is currently being investigated in clinical trials recruiting KRAS-altered tumors (clinicaltrials.gov).

Binimetinib — **MEK Inhibitor** — ***KRAS***

Binimetinib (Mektovi) is a MEK inhibitor, FDA-approved in combination with encorafenib, for the treatment of patients with unresectable or metastatic melanoma with a BRAF V600E or V600K mutation, detected by an FDA-approved test (Mektovi [package insert], Array BioPharma; accessed May 2019). Binimetinib is currently being investigated in clinical trials recruiting KRAS-altered tumors (clinicaltrials.gov).

Cobimetinib — **MEK Inhibitor** — ***KRAS***

Cobimetinib (Cotellic) is a MEK Inhibitor, FDA approved in combination with vemurafenib (Zelboraf) for BRAF V600E/K unresectable or metastatic melanoma (Cotellic [package insert]. Genentech; 2018; accessed July 2019). Cobimetinib is currently being investigated in clinical trials recruiting KRAS-altered tumors (clinicaltrials.gov).

Neratinib — **HER Inhibitor** — ***KRAS***

Page 2 of 6

S5. Example of genomic tumor test report (Page 3 of 6)

The Jackson Laboratory
Leading the search for tomorrow's cures

Patient Name: | **Test:** ActionSeq™ 2.0 Plus
Patient ID: | **Report Date:** 2019

Neratinib (Nerlynx) is a tyrosine kinase inhibitor (TKI), FDA approved for the extended adjuvant therapy of adults with early stage HER2-overexpressed/amplified breast cancer, following adjuvant trastuzumab-based therapy (Neratinib (Nerlynx). Puma Biotechnology; 2018 [cited 2019 May]). Neratinib is currently being investigated in clinical trials recruiting KRAS-altered tumors (clinicaltrials.gov).

Talazoparib | **PARP Inhibitor** | ***KRAS***

Talazoparib (Talzenna) is a poly (ADP-ribose) polymerase (PARP) inhibitor FDA-approved for the treatment of adult patients with deleterious or suspected deleterious germline BRCA-mutated HER2-negative locally advanced or metastatic breast cancer (Talazoparib [package insert]. Pfizer, 2018 [cited Apr, 2019]). Talazoparib is currently being investigated in clinical trials recruiting KRAS-altered tumors (clinicaltrials.gov).

Trametinib | **MEK Inhibitor** | ***KRAS***

The MEK1/2 inhibitor Trametinib (Mekinist) is FDA approved as monotherapy for BRAF V600E/K unresectable or metastatic melanoma, and is FDA approved in combination with Dabrafenib (Tafinlar) for the following indications: BRAF V600E/K unresectable or metastatic melanoma. Adjuvant treatment of BRAF V600E/K melanoma with involvement of lymph node(s), following complete resection. BRAF V600E metastatic non-small cell lung cancer (NSCLC). Locally advanced or metastatic anaplastic thyroid cancer (ATC) with BRAF V600E mutation and with no satisfactory locoregional treatment options. Note: Trametinib is not indicated for patients with melanoma who have progressed on prior BRAF-inhibitor therapy (Mekinist [package insert]. Novartis; 2018 [cited 2019 May]). Trametinib is currently being investigated in clinical trials recruiting KRAS-altered tumors (clinicaltrials.gov).

Temsirolimus | **mTOR Inhibitor** | ***PIK3CA***

Temsirolimus (Torisel) is a kinase inhibitor that targets the mammalian target of rapamycin (mTOR). Temsirolimus is FDA approved for advanced renal cell carcinoma (Torisel [package insert]. Pfizer; 2015 [cited April 2019]). Temsirolimus is currently being investigated in clinical trials recruiting PIK3CA-altered tumors (clinicaltrials.gov).

Therapies with Resistance Guidelines

None | **No therapies with resistance or contraindication guidelines were identified**

Potential Clinical Trials

Gene	Phase	Drug	Drug Class	Trial ID	Title
KRAS	Phase II	AZD1775	WEE1 Inhibitor	NCT02576444	OLAParib COmbinations
KRAS	Phase II	Avelumab<br>Binimetinib<br>Talazoparib	Anti-PD-1 mAb<br>MEK Inhibitor<br>PARP Inhibitor	NCT03637491	A Study of Avelumab, Binimetinib and Talazoparib in Patients With Locally Advanced or Metastatic RAS-mutant Solid Tumors
PIK3CA	Phase II	AZD5363	AKT Inhibitor	NCT02576444	OLAParib COmbinations
PIK3CA	Phase II	Ipatasertib	AKT Inhibitor	NCT03337724	A Study of Ipatasertib in Combination With Paclitaxel as a Treatment for Participants With PIK3CA/AKT1/PTEN-Altered, Locally Advanced or Metastatic, Triple-Negative Breast Cancer or Hormone Receptor-Positive, HER2-Negative Breast Cancer (IPATunity130)
PIK3CA	Phase II	Temsirolimus	mTOR Inhibitor	NCT03297606	Canadian Profiling and Targeted Agent Utilization Trial (CAPTUR)
PIK3CA	Phase II	Alpelisib	PI3K Inhibitor	NCT03056755	Study Assessing the Efficacy and Safety of Alpelisib Plus Fulvestrant or Letrozole, Based on Prior Endocrine Therapy, in Patients With PIK3CA Mutation With Advanced Breast Cancer Who Have Progressed on or After Prior Treatments
PIK3CA	Phase II	Orteronel	Androgen Synthesis Inhibitor	NCT01990209	Orteronel as Monotherapy in Patients With Metastatic Breast Cancer (MBC) That Expresses the Androgen Receptor (AR)
PIK3CA	Phase II	Palbociclib	CDK4/6 Inhibitor	NCT01723774	PD 0332991 and Anastrozole for Stage 2 or 3 Estrogen Receptor Positive and HER2 Negative Breast Cancer
KRAS	Phase I/Phase II	Trametinib	MEK Inhibitor	NCT02079740	Trametinib and Navitoclax in Treating Patients With Advanced or Metastatic Solid Tumors
KRAS	Phase I/Phase II	ASTX029	ERK Inhibitor	NCT03520075	Study of ASTX029 in Subjects With Advanced Solid Tumors
KRAS	Phase I/Phase II	Neratinib	HER Inhibitor	NCT03919292	Neratinib + Valproate in Advanced Solid Tumors, w/Expansion Cohort in Ras-Mutated Ca
KRAS	Phase I/Phase II	Cobimetinib<br>RMC-4630	MEK Inhibitor<br>SHP2 Inhibitor	NCT03989115	Dose-Escalation and Dose-Expansion of RMC-4630 and Cobimetinib in Relapsed/Refractory Solid Tumors
KRAS	Phase I	LXH254	RAF Inhibitor	NCT02607813	Phase I Study of LXH254 in Patients With Advanced Solid Tumors Haboring MAPK Pathway Alterations
KRAS	Phase I	KO-947	ERK Inhibitor	NCT03051035	First-in-Human Study of KO-947 in Non-Hematological Malignancies
KRAS	Phase I	Olaparib<br>Selumetinib	PARP Inhibitor<br>MEK Inhibitor	NCT03162627	Selumetinib and Olaparib in Solid Tumors
KRAS	Phase I	Neratinib<br>Trametinib	HER Inhibitor<br>MEK Inhibitor	NCT03065387	Study of the Pan-ERBB Inhibitor Neratinib Given in Combination With Everolimus, Palbociclib or Trametinib in Advanced Cancer Subjects With EGFR Mutation/Amplification, HER2 Mutation/Amplification, HER3/4

S5. Example of genomic tumor test report (Page 4 of 6)

The Jackson Laboratory
Leading the search for tomorrow's cures

Patient Name:
Patient ID:
Test: ActionSeq™ 2.0 Plus
Report Date: 2019

					Mutation or KRAS Mutation
KRAS	Phase I	RMC-4630	SHP2 Inhibitor	NCT03634982	Dose Escalation of RMC-4630 Monotherapy in Relapsed/Refractory Solid Tumors
KRAS	Phase I	TAK-659	TRK Inhibitor	NCT03756818	TAK-659 and Paclitaxel in Treating Patients With Advanced Solid Tumors
PIK3CA	Phase I	ARQ751	AKT Inhibitor	NCT02761694	ARQ 751 as a Single Agent or in Combination With Other Anti-Cancer Agents, in Solid Tumors With PIK3CA / AKT / PTEN Mutations
PIK3CA	Phase I	GDC-0077	PI3K Inhibitor	NCT03006172	To Evaluate the Safety, Tolerability, and Pharmacokinetics of GDC-0077 Single Agent in Participants With Solid Tumors and in Combination With Endocrine and Targeted Therapies in Participants With Breast Cancer
PIK3CA	Phase I	Gedatolisib<br>Palbociclib	PI3K Inhibitor<br>CDK4/6 Inhibitor	NCT03065062	Study of the CDK4/6 Inhibitor Palbociclib (PD-0332991) in Combination With the PI3K/mTOR Inhibitor Gedatolisib (PF-05212384) for Patients With Advanced Squamous Cell Lung, Pancreatic, Head & Neck and Other Solid Tumors

Note: Only clinical trials specifically involving the genes or genomic alterations identified in the patient are reported unless otherwise mentioned. Reporting of the NCI-MATCH trial(NCT02465060) only assumes a reasonable gene - disease association has been identified and the patient is therefore potentially eligible. Other conditions apply. If the patient meets all criteria under the NCI laboratory testing protocol, an official letter will be sent to the ordering physician. For additional criteria regarding clinical trials listed within this report, we suggest searching the trial identifier at ClinicalTrials.gov.

Variants of Unknown Clinical Significance (VUS): Tier III

Gene	Variant	Coordinate	Protein Effect	AF/Depth	COSMIC ID	Functional Impact
APC NM_000038.5	p.A2274V c.6821C>T	chr5:112842415	Missense	51.0%/1220X	N/A	Unknown
CDKN2C NM_001262.2	p.G48V c.143G>T	chr1:50973906	Missense	63.0%/719X	N/A	Unknown
FLCN NM_144997.5	p.V151L c.451G>T	chr17:17224089	Missense	13.0%/940X	N/A	Unknown
KMT2D NM_003482.3	p.P3665A c.10993C>G	chr12:49033712	Missense	50.0%/1725X	N/A	Unknown
PIK3CA NM_006218.2	p.E600K c.1798G>A	chr3:179219622	Missense	37.0%/825X	COSM1537052	Unknown
WRN NM_000553.4	p.T1262R c.3785C>G	chr8:31154721	Missense	11.0%/232X	N/A	Unknown

Test Methods & Limitations

ActionSeq™ 2.0 Plus incorporates two targeted-enrichment sequencing assays: a DNA-based panel (ActionSeq™ 2.0) comprising 501 cancer-related genes for which all coding exons are sequenced and clinically significant variants in 209 genes are reported, and a RNA-based panel (FusionSeq™ 2.0) evaluating the transcriptome for 548 genes known to form fusions in solid tumors and reporting clinically significant fusions across 53 gene partners.

The ActionSeq™ 2.0 uses genomic DNA extracted from macro dissection-enriched FFPE tissue sections (≥30% neoplastic content), followed by enrichment of target exons and introns by hybrid-capture (Agilent Technologies). Illumina sequencers generated 149bp paired-end sequence reads with a mean coverage of greater than or equal to 500X. A minimum coverage of 50X was required for reporting SNVs (single nucleotide variants) and indels (insertions and deletions up to 50-bp in length). Variants within regions that do not meet our coverage thresholds are not reported. For a list of these regions, please contact jaxmoleculardx@jax.org. The LOD (limit of detection) for SNVs and indels was determined as 5%. The LOD for copy number variants (CNVs) was 5 copies for amplifications and 1 copy for deletions. Mutational analysis was performed using the ActionSeq™ 2.0 Genome Analytics (AGA2) pipeline, developed at The Jackson Laboratory (JAX), linking an in-house QC Toolkit, fastp, BWA, Picard, UMI-tools, GATK tool kit, CONTRA, and Control-FREEC for QC, alignment, and variant discovery. Variants were called against human genome build GRCh38.

The FusionSeq™ 2.0 assay uses total RNA extracted from macro dissection-enriched FFPE tissue sections, followed by cDNA synthesis, and subsequent library amplification (KAPA Biosystems). Illumina sequencers generated 75bp paired-end sequence reads with a minimum of 40 million reads per sample. The detection limit for in-frame fusion variant calls is ≥5 spanning unique reads. Fusion calls <5 unique spanning reads deemed clinically actionable are confirmed using Taqman RT-PCR or re-running samples before being included in the clinical report. Fusion analysis is performed using an in-house developed fusion caller, which aligns against the hg19 human genome build.

Evidence of association between genomic variants and potential therapeutic (including clinical trials), prognostic and/or diagnostic outcomes is obtained from peer-reviewed literature, clinical practice guidelines, FDA labels, publically available databases and the JAX Clinical Knowledgebase (CKB). Information from these sources is curated into Variant Explorer (VariEx; Precision Health Software, Inc), the JAX Clinical Laboratory's patient knowledgebase and clinical significance of genomic variants interpreted in the context of each patient's molecular/disease profile The ActionSeq™ 2.0 Plus report reflects the variants determined to be clinically relevant at the time of reporting. Variants are classified into four tiers based on the joint consensus guidelines published by AMP/ASCO/CAP on interpretation of sequence variants in cancer (PMID: 27993330). The four tiers include strong clinical significance (Tier I), potential clinical significance (Tier II), unknown clinical significance (Tier III) and benign or likely benign variants (Tier IV). The patient's complete molecular profile is available to the ordering clinician(s) upon request, up to 18 months after date of report, including allele frequencies for variants of uncertain significance (VUS) and variants with no current therapeutic correlation. These variants are included and made available to clinicians, upon request, so they may act in the event therapeutic options or other clinical evidence emerge.

Tumor mutation burden (TMB) is calculated as the mutations per megabase (mut/Mb) across the ~2.3Mb of coding DNA captured by the ActionSeq™ 2.0 panel. Tumors containing ≥20 mut/Mb are classified as TMB-high and may respond to immunotherapy treatment (PMID: 28835386). Microsatellite instability (MSI) is reported as stable (MSS) or high (MSI-H) and calculated as the total frameshift load (fs/Mb) combined with the tumor mutational burden (mut/Mb) for ~2.3Mb of coding DNA captured by the ActionSeq™ 2.0 panel. MSI status is reported according to the following cutoff criteria: MSI-H, ≥2.6 fs/Mb AND ≥8.5 mut/Mb; MSS, <2.6 fs/Mb OR <8.5 mut/Mb.

S5. Example of genomic tumor test report (Page 5 of 6)

The Jackson Laboratory
Leading the search for tomorrow's cures

Patient Name: | **Test:** ActionSeq™ 2.0 Plus
Patient ID: | **Report Date:** 2019

Disclaimer

Decisions on patient care must be based on the independent medical judgment of the treating physician, taking into consideration all relevant information about the patient's condition, including patient and family history, physical examinations, information from other diagnostic tests, and patient preferences. A treating physician's decisions should not be based on a single test, such as this test, or the information contained in this report alone. Results of this test must always be interpreted in the context of all relevant clinical and pathological data, and should not be used alone for diagnosis or patient care decisions.

ActionSeq™ 2.0 Plus uses high throughput sequencing to identify clinically significant variants (SNVs, indels and CNVs) within 209 cancer-related genes and fusions across 53 gene partners as listed in the appendix of this report. The assay may not detect all potentially relevant variants. Tumor tissue is not homogenous and its characteristics may differ from sample to sample for the same tumor. It may be possible for a biomarker variant to be present, yet go undetected by our assay either due to the heterogeneous nature of the tumor tissue or the limit of detection of our assay (please see "test methods and limitations" section). Therefore, to the extent a particular biomarker variant is not reported, we cannot guarantee that the variant does not exist.

ActionSeq™ 2.0 Plus examines tumor tissue only and does not examine normal tissue (such as tissue adjacent to the tumor). Thus, the origin of a mutation detected by our assay may be a somatic (not inherited) or a germline mutation (inherited) and will not be distinguishable by this assay. Germline variants are not analyzed or reported. If a germline inheritance pattern is suspected, then counseling by a genetic counselor is recommended.

The information presented in the clinical trials section of this report is compiled from public sources believed to be reliable and current. However, the information available in the public domain is continuously updated. While we endeavor to make this information accurate and complete, we cannot guarantee the accuracy or completeness of this information. Accordingly, the patient's physician or research staff should independently investigate the clinical trials information. The clinical trials information was compiled from www.clinicaltrials.gov. The clinical trials are not ranked in order of potential or predicted efficacy. The clinical trial information is to be used for clinical trial guidance and may not include all relevant trials. The clinical trials listed in this report were enrolling at the time of report generation, but the status may change at any time. Specific entrance criteria for each clinical trial should be reviewed as additional inclusion criteria may apply. The clinical trials identified may or may not be suitable for a particular patient and we do not guarantee or suggest that any particular trial will be effective with the treatment of any particular condition. Health care providers should employ independent clinical judgment in interpreting this information for their patients.

This report includes information about therapeutic agents that appear to be associated with clinical benefit based on National Comprehensive Cancer Network (NCCN) Compendium guidelines, relevance of tumor lineage, and published evidence, as available and compiled by The Jackson Laboratory. The Jackson Laboratory expressly disclaims and makes no representation or warranty relating to the published evidence and scientific literature identified in this report, or any of the conclusions and information set forth in this report that is derived from a review thereof, including information and conclusions relating to therapeutic agents that are included or omitted from this report. The therapeutic agents included in this report are not ranked in order of potential or predicted efficacy. Agents with potential clinical benefit (or lack of clinical benefit) are not evaluated for source or level of published evidence, and are identified based on the information available at the time of the test. The agents identified may or may not be suitable for use with a particular patient and we do not guarantee or suggest that any particular agent will be effective with the treatment of any particular condition. The selection of any, all or none of the agents associated with potential clinical benefit (or lack of clinical benefit) resides solely within the discretion of the treating physician.

This report includes some clinically relevant interpretation of next-generation sequencing data powered by The Jackson Laboratory Clinical Knowledgebase (CKB). This information may include associations between a biomarker variant (or lack of a variant) and one or more therapeutic agents with potential clinical benefit (or lack of clinical benefit), including agents that are being studied in clinical research. A finding of a biomarker variant does not necessarily indicate pharmacologic effectiveness (or lack thereof) of any agent or treatment regimen. A finding of "no biomarker variant" does not necessarily indicate lack of pharmacologic effectiveness (or lack of effectiveness) of any agent or treatment regimen. The Jackson Laboratory expressly disclaims, and makes no representation or warranty of, the accuracy or completeness with respect to the publicly available information included herein or complied in creating this report.

This test was developed and its performance characteristics determined by The Jackson Laboratory. It has not been cleared or approved by the U.S. Food and Drug Administration (FDA). This test may be used for clinical purposes and should not be regarded as purely investigational or for research only. This laboratory is certified under the Clinical Laboratory Improvement Amendments of 1988 (CLIA-88) as qualified to perform high complexity clinical testing. The Jackson Laboratory makes no promises or guarantees that a healthcare provider, insurer or other third party payor, whether private or governmental, will reimburse a patient for the cost of this test.

Appendix I – Additional Genomic Information for Clinically Significant Variant(s)

Variant	Transcript	Genomic Coordinate	Genome Build
KRAS p.G12A	NM_033360.3	chr12:25245350	Hg38
PIK3CA p.H1047R	NM_006218.2	chr3:179234297	Hg38

Appendix IIa – Genes Sequenced on the JAX ActionSeq™ 2.0 Panel

A1CF, **ABL1, ABL2, ABRAXAS1(FAM175A),** ACVR1B, ADAR, ADARB1, AFDN(MLLT4), AFF2, AGO1, AGO2, AGO3, AGO4, AICDA, AIM2, **AKT1, AKT2, AKT3, ALK,** AMER1, **APC,** APOBEC1, APOBEC2, APOBEC3A, APOBEC3C, APOBEC3D, APOBEC3F, APOBEC3G, APOBEC4, **AR, ARAF, ARID1A, ARID1B, ARID2,** ASH1L, ASXL1, **ATM,** ATN1, ATP10B, **ATR, ATRX, AURKA, AURKB, AURKC, AXIN1, AXL,** B2M, **BAP1, BARD1, BCL2,** BCL2L1, BCOR, BCR, BID, **BIRC2, BIRC3, BIRC5, BLM, BRAF, BRCA1, BRCA2,** BRD3, **BRD4, BRIP1,** BTK, CALR, CARD11, CARM1, CASP8, CBFB, **CBL, CCND1, CCND2, CCND3, CCNE1,** CCR2, **CD274,** CD74, CD79A, CD79B, CDC73, **CDH1, CDK12, CDK4, CDK6, CDK8, CDKN1A, CDKN1B, CDKN2A, CDKN2C,** CEBPA, **CHEK1, CHEK2,** CIC, CIRBP, COL18A1, **CREBBP, CRKL,** CRLF2, **CRTC1, CRTC2, CSF1R,** CSF3R, CSMD3, CTCF, CTLA4, **CTNNB1,** CUX1, CXCR4, CYLD, DALRD3, DAXX, DDIT4, DDR1, **DDR2,** DDX11, DICER1, DNMT1, DNMT3A, DNMT3B, DOT1L, DROSHA, DSPP, DYRK1B, **EGFR,** EHMT2, **EP300,** EPHA10, **EPHA2,** EPHA3, EPHA5, **EPHB4,** EPHB6, **ERBB2, ERBB3, ERBB4, ERCC2, ERCC3,** ERG, **ESR1,** ETV1, ETV6, EZH1, **EZH2,** FAM166A, **FANCA, FANCC, FANCD2, FANCE, FANCF, FANCG, FANCL, FANCM,** FAT4, FBXO4, **FBXW7,** FER1L5, FES, **FGF19, FGF3, FGFR1, FGFR2, FGFR3, FGFR4, FH, FLCN, FLT1, FLT3, FLT4,** FOXA1, FOXL2, FRK, FRS2, FUBP1, GATA1, GATA2, GATA3, **GEN1, GLI1, GLI2, GLI3,** GLI4, **GNA11,** GNAO1, **GNAQ, GNAS,** GPR32, GPRIN2, GPS2, GRIN2A, GRM3, H2AFX, H3F3A, HAUS5, HCFC1R1, **HDAC2,** HES1, HES5, HEY1, HEY2, **HGF,** HIF1A, HIST1H1C, HIST1H2BC, HIST1H3B, HLA-A, HMCN1, HNF1A, **HRAS,** HSBP1, HSF1, HSF2, HSF4, HSP90AA1, HSP90AB1, **ID1,** ID2, ID3, ID4, **IDH1, IDH2, IGF1R,** IKBKE, IKZF1, IL17RB, IL7R, INHBA, INO80C, **IRS2,** ITGB3, ITPKB, **JAK1, JAK2, JAK3,** JMJD1C, JMJD6, **JUN,** KCNQ2, KDM1A, KDM1B, KDM2A, KDM2B, KDM3A, KDM3B, KDM4A, KDM4B, **KDM4C,** KDM4D, KDM5A, KDM5B, KDM5C, KDM5D, **KDM6A,** KDM6B, **KDR, KEAP1,** KIAA1524, **KIT,** KLF4, KLHL6, KLRG2, KMT2A, **KMT2C, KMT2D, KRAS,** LATS1, LATS2, LCK, LLGL2, **LMO1,** LRFN4, **LRP1B,** LTK, LYSMD3, **MAP2K1, MAP2K2,** MAP2K4, MAP2K7, MAP3K1, MAP3K9, **MAPK1,** MAPK3, MAPK8, MAPK9, **MCL1, MDM2, MDM4,** MED12, MED23, **MEN1, MET, MLH1, MLH3,** MPL, **MRE11(MRE11A), MSH2, MSH3, MSH6,** MST1, **MST1R, MTOR,** MUC16, MUC17, MUC4,

S5. Example of genomic tumor test report (Page 6 of 6)

The Jackson Laboratory
Leading the search for tomorrow's cures

Patient Name:
Patient ID:
Test: ActionSeq™ 2.0 Plus
Report Date: 2019

*MUC5B, **MUTYH,** MYB, **MYC, MYCL, MYCN,** MYD88, MYT1L, **NBN, NCOA3,** NCOR1, NEK10, **NF1, NF2, NFE2L2,** NFKB1, NISCH, NKAIN4, NKX2-1, **NOTCH1, NOTCH2, NOTCH3,** NOTCH4, NPM1, **NRAS,** NSD1, NSD2(WHSC1), NSD3(WHSC1L1), **NTRK1, NTRK2, NTRK3,** NUAK2, **PAK1,** PAK3, **PALB2,** PARP1, PAX5, **PBRM1, PDGFA,** PDGFB, **PDGFRA, PDGFRB, PDPK1,** PGR, PHF6, PIK3C2B, **PIK3CA, PIK3CB, PIK3CD, PIK3CG, PIK3R1, PIK3R2, PIM1,** PIP4K2C, PIWIL1, PLK1, **PMS1, PMS2, POLB,** POLI, POLR2A, PPARG, PPEF1, **PPM1D,** PPP2R1A, PPP2R1B, PQLC2, **PRDM1,** PRDM14, PRDM2, PRDM9, PRKAA1, PRKAA2, PRMT1, PRMT2, PRMT3, PRMT5, PRMT6, PRRX1, **PTCH1, PTEN,** PTK2, PTK2B, **PTPN11,** PTPRB, PTPRD, PYCR3(PYCRL), RAC1, **RAD50, RAD51, RAD51B, RAD51C, RAD51D, RAD52,** RAD54B, **RAD54L,** RAD54L2, RAF1, RARA, RASA3, RASGRF2, **RB1, RET, RHEB, RICTOR,** RIT1, **RNF43,** ROR2, **ROS1, RPA1,** RPGR, RPS6KB1, RPTOR, RSPO2, RSPO3, RUNX1, SAAL1, SCGB1C1, SDHA, **SDHB,** SDHC, SDHD, SEM1(SHFM1), SETBP1, SETD2, SETD7, SF3B1, SIRT4, SKP2, SLC38A3, **SLX4,** SMAD2, **SMAD4, SMARCA4, SMARCB1, SMARCC1, SMO,** SMYD3, SOCS1, SOS1, SOX9, SPOP, **SRC,** SRSF2, STAG2, **STAT3, STAT5A, STAT5B, STK11,** STK3, SUV39H1, **SYK,** TARBP2, TBL1XR1, TBP, TBX3, TCF7L2, TEAD1, TEAD2, TEAD4, TEK, TERT, TET2, TGFB1, **TGFBR2,** THBS1, TLE6, TMEM82, TNFAIP3, **TNK2, TP53,** TP63, TP73, TRAF7, **TSC1, TSC2,** TSHR, TTC5, TTK, TTN, U2AF1, UBC, USH2A, VEGFA, **VHL,** WEE1, WNT1, WNT7A, **WRN,** WT1, WWTR1, **XRCC2, XRCC3, YAP1, YES1,** ZFP36L1, ZMYND19*

Regions of low coverage or no coverage are available upon request. Clinically significant variants are reported only in bolded genes.

Appendix IIb – Genes Sequenced on the JAX FusionSeq™ 2.0 Panel

*ABCC4, ABI1, **ABL1,** ABL2, ACACA, ACE, ACER1, ACKR3, ACSL6, ADD3, AFF1, AFF3, AFF4, AGR3, AHI1, AHRR, AKAP12, AKT3, **ALK,** ANKRD28, AR, ARHGAP20, ARHGAP26, ARNT, ASPSCR1, ASTN2, **ATF1,** ATIC, ATP1B4, AUTS2, **AXL,** BACH2, BAG4, BAIAP2L1, BAZ2A, BCAS3, BCAS4, BCL10, BCL11A, BCL11B, **BCL2,** BCL2L1, BCL3, **BCL6,** BCL9, BCOR, **BCR,** BDNF, BICC1, BIRC3, BIRC6, **BRAF, BRCA1, BRCA2,** BRD1, BRD3, BRD4, BRWD3, BTBD18, BTG1, C11orf1, C11orf95, C2CD2L, C3orf27, CAMTA1, CAPRIN1, CARS, CASC5, CASP7, CBFA2T3, **CBFB,** CBL, CCAR2, CCDC28A, CCDC6, CCDC88C, CCNB1IP1, CCNB3, CCND1, CCND2, CCND3, **CD74,** CDH11, CDK5RAP2, CDK6, CDX1, CDX2, CEBPA, CEBPB, CEBPD, CEBPE, CEP170B, CEP85L, CHD6, CHIC2, CHMP2B, CHST11, CIC, CIITA, CLP1, CLTC, CLTCL1, CMKLR1, CNBP, CNOT2, CNTRL, COG5, COL1A1, COL1A2, COL6A3, COX6C, CPSF6, CRADD, CREB1, CREB3L1, CREB3L2, CREBBP, CRLF2, CRTC1, CSF1, CSF1R, CTDSP2, CTNNB1, CUX1, DAB2IP, DACH1, DACH2, DDIT3, DDX10, DDX20, DEK, DMRT1, DNAJB1, DNASE2, DPM1, DUSP22, DUX4, EBF1, EEFSEC, **EGFR,** EGR1, EGR2, EGR3, EGR4, **EIF3E,** EIF4A2, ELF4, ELK4, ELL, ELN, EML1, **EML4,** EP300, EP400, EPC1, EPOR, EPS15, **ERBB2,** ERBB3, ERC1, ERCC1, **ERG,** ERLIN2, **ESR1,** ETS1, ETV1, **ETV4,** ETV5, ETV6, **EWSR1,** EZR, FAM19A2, FCGR2B, FCRL4, FEN1, FEV, FGF8, **FGFR1,** FGFR1OP, FGFR1OP2, **FGFR2, FGFR3, FGFR4,** FGR, FHIT, **FIP1L1, FLI1,** FLNA, FLT3, FLT3LG, FNBP1, FOSB, FOSL1, FOXO1, FOXO4, FOXP1, FRK, FRYL, FUS, GAS5, GAS7, GATA1, GIT2, GLI1, GORASP2, GOSR1, GOT1, GPR107, GPR128, GPR34, GRHPR, GRID1, GTF2I, H2AFX, HAS2, HEY1, HHEX, HIP1, HIPK1, HIST1H4I, HLF, HMGA2, HNF1A, HOXA10, HOXA11, HOXA13, HOXA9, HOXC11, HOXC13, HOXD11, HOXD13, HSP90AA1, ID4, IKZF1, IL2, IL21R, IL3, INPP5D, INSR, IQCG, IRF2BP2, IRF4, IRS4, ITK, JAK1, JAK2, JAZF1, KANK1, KAT6A, KAT6B, KDM5A, KIAA1524, KIF5B, **KMT2A,** KPNB1, KSR1, LASP1, LCK, LCP1, LGR5, LHFP, LHX2, LHX4, LINC00598, LINC00982, LMBRD1, LMO1, LMO2, LNP1, LPP, LPXN, LRMP, LRRC37B, LTBP1, LYL1, MACROD1, MAF, MAFB, MALT1, MAML2, MAPRE1, **MAST1,** MAST2, MBNL1, MBTD1, MDS2, MEAF6, MECOM, **MET,** MGEA5, MKL1, MKL2, MLF1, MLLT1, MLLT10, MLLT11, MLLT3, MLLT4, MLLT6, MN1, MNX1, MSI2, MSMB, MSN, MTHFD1L, MUC1, MUSK, MUTYH, MYB, MYBL1, **MYC, MYH11,** MYH9, MYO18A, MYO1F, NAB2, NAPA, NBEAP1, NBR1, NCOA1, NCOA2, NCOA3, NCOA4, NDE1, NF1, NFATC2, NFIB, **NFKB2,** NGF, NGFR, NIN, NIPBL, NKX2-5, NONO, **NOTCH1,** NOTCH2, NPM1, NR4A3, NR6A1, **NRG1,** NSD1, NT5C2, NTF3, NTF4, **NTRK1, NTRK2, NTRK3,** NUMA1, NUMBL, NUP107, NUP214, NUP98, NUTM1, NUTM2A, NUTM2B, OFD1, OLIG2, OLR1, OMD, P2RY8, PAPPA, PATZ1, PAX3, PAX5, PAX7, **PAX8,** PBX1, PCM1, PDE4DIP, PDGFB, **PDGFRA, PDGFRB,** PER1, PHF1, PHF23, PHIP, PICALM, PIK3CA, PIM1, PKM, PKN1, PLAG1, **PML,** POM121, POU2AF1, POU5F1, PPAP2B, **PPARG,** PPARGC1A, PPFIBP1, PPP2R1B, PRCC, PRDM16, PRKACA, PRKAR1A, PRKCA, PRKCB, PRKG2, PRRX2, PSIP1, PSMD2, **PTPRK,** PTPRR, PVT1, **QKI,** RABEP1, RAD51B, **RAF1,** RANBP2, RAP1GDS1, **RARA,** RBM15, RBM6, RCOR1, RCSD1, RELA, **RET,** RHOH, RNF213, **ROS1,** RPL22, RPN1, RREB1, RRM1, **RSPO2, RSPO3,** RTEL1, **RUNX1,** RUNX1T1, SARNP, SEC31A, SEPT2, SEPT5, SEPT6, SEPT9, SERPINE1, SERPINF1, SET, SETBP1, SFPQ, SH3D19, SH3GL1, SIK3, SLC34A2, SLC45A3, SLCO1B3, SMAP1, SMARCA5, SMARCB1, SNHG5, SORBS2, SORT1, SP3, SPDYE4, SPECC1, SPTBN1, SQSTM1, SRF, SRSF3, SS18, SS18L1, SSBP2, SSX1, SSX2, SSX4, ST6GAL1, STAT5B, STAT6, STRN, SUGP2, SUZ12, SYK, TACC1, TACC2, TACC3, TAF15, TAF6L, TAL1, TAL2, TAOK1, TBX15, TCF12, TCF3, TCL1A, TCTA, TEAD1, TEAD2, TEAD3, TEAD4, TEC, TENM1, **TERT,** TET1, TFE3, TFEB, TFG, TFPT, TFRC, TGFBR3, THADA, THRAP3, TIRAP, TLX1, TLX3, TMEM66, **TMPRSS2,** TNFRSF17, TOP1, TOP2B, TP53BP1, TPM3, TPM4, TRHDE, TRIM24, TRIP11, TRPS1, USP16, USP42, USP6, VGLL3, WASF2, WDR18, WDR70, WHSC1, WHSC1L1, WSB1, WT1, WWTR1, XIAP, YAP1, YTHDF2, YWHAE, ZBTB16, ZC3H7A, ZC3H7B, ZFP64, ZFPM2, ZFYVE19, ZMIZ1, ZMYM2, ZMYND11, ZNF207, ZNF384, ZNF444, ZNF521, ZNF585B, ZNF687, ZNF84*

Regions of low coverage or no coverage are available upon request. Clinically significant variants are reported only in bolded genes.

Name: ______________________________ **Date:** ____________________

Clinical Laboratory Director

S6. Supplemental references

1. Rueter, J., Anderson, E. C., Graham, L. C., Antov, A., Helbig, P., Gaitor, L., Bourne, J., Edelman, E., Reed, E. K., Reddi, H. V., Mockus, S., DiPalazzo, J., Lu-Emerson, C., Inhorn, R., Sinclair, S. J., Thomas, C. A., Brooks, P. L., Rasmussen, K., Han, P., … Group, M. W. (2023). The Maine Cancer Genomics Initiative: Implementing a Community Cancer Genomics Program Across an Entire Rural State. *JCO Precision Oncology*. https://doi.org/10.1200/PO.22.00619
2. Cromartie, J. (USDA). (n.d.). *Rural-Urban Commuting Area (RUCA) Codes*. Retrieved November 12, 2020, from https://www.ers.usda.gov/data-products/rural-urban-commuting-area-codes/documentation/

S7. Supplementary Note

Members of the MCGI Working Group

Maine Cancer Genomics Initiative Steering Committee Members

Central Maine Medical Center, Lewiston: Nicholette Erickson; Dahl-Case Pathology Associates: Mayur Movalia, Marek Skacel; Jefferson Cary Cancer Center, Caribou: Allan Espinosa; MaineGeneral Medical Center, Augusta: Ridhi Gupta, Rachit Kumar, Richard Polkinghorn; Maine Medical Center, Portland: Christopher Darus, Scot Remick; Maine Medical Center, Spectrum Medical Group, Portland: Robert Christman, Karen Rasmussen; New England Cancer Specialists, Scarborough: Christian Thomas; Northern Light Eastern Maine Medical Center, Bangor: Philip Brooks, Catherine Chodkiewicz, Antoine Harb, Sarah Sinclair; Southern Maine Health Care Biddeford: Peter Rubin: Waldo County General Hospital, Belfast: Elizabeth Connelly; York Hospital, York: Peter Georges; The Jackson Laboratory: Jennifer Bourne, Linda Choquette, Ken Fasman, Cristen Flewellen, Emily Edelman, Lory Guerrette, Petra Helbig, Susan Mockus, Kate Reed, Jens Rueter, Kunal Sanghavi.

Center for Interdisciplinary Population & Health Research, MaineHealth Institute for Research

Eric Anderson, Sumayo Awale, Jessica DiBiase, John DiPalazzo, Anny Fenton, Caitlin Gutheil, Paul Han, Ally Hinton, Michael Kohut, Susan Leeds, Lee Lucas, Elizabeth Scharnetzki, Leo Waterston, Lisbeth Wierda.
